# The impact of environmental changes on the yield and nutritional quality of fruits, nuts and seeds: a systematic review

**DOI:** 10.1088/1748-9326/ab5cc0

**Published:** 2019-11-28

**Authors:** Carmelia Alae-Carew, Salina Nicoleau, Frances A Bird, Poppy Hawkins, Hanna L. Tuomisto, Andy Haines, Alan D. Dangour, Pauline F.D. Scheelbeek

**Affiliations:** 1 Department of Population Health, London School of Hygiene & Tropical Medicine, London, WC1E 7HT, UK; 2 Centre on Climate Change and Planetary Health, London School of Hygiene & Tropical Medicine, London, WC1E 7HT, UK; 3 Department of Agricultural Sciences, University of Helsinki, Helsinki, 00100, Finland; 4 Department of Public Health, Environments and Society, London School of Hygiene & Tropical Medicine, London, WC1E 7HT, UK

**Keywords:** Fruits, Nuts, Seeds, Yields, Environmental change, Environmental exposure

## Abstract

**Background:**

Environmental changes are predicted to threaten human health, agricultural production and food security. Whilst their impact has been evaluated for major cereals, legumes and vegetables, no systematic evidence synthesis has been performed to date evaluating impact of environmental change on fruits, nuts and seeds (FN&S) - valuable sources of nutrients and pivotal in reducing risks of non-communicable disease.

**Methods:**

We systematically searched seven databases, identifying available published literature (1970-2018) evaluating impacts of water availability and salinity, temperature, carbon dioxide (CO2) and ozone (O3) on yields and nutritional quality of FN&S. Dose-response relationships were assessed and, where possible, mean yield changes relative to baseline conditions were calculated.

**Results:**

81 papers on fruits and 24 papers on nuts and seeds were identified, detailing 582 and 167 experiments respectively. A 50% reduction in water availability and a 3-4dS/m increase in water salinity resulted in significant fruit yield reductions (mean yield changes: -20.7% [95%CI -43.1% to -1.7%]; and -28.2% [95%CI -53.0% to -3.4%] respectively). A 75-100% increase in CO2 concentrations resulted in positive yield impacts (+37.8%; [95%CI 4.1% to 71.5%]; and 10.1%; [95%CI -30.0% to 50.3%] for fruits and nuts respectively). Evidence on yield impacts of increased O3 concentrations and elevated temperatures (>25°C) was scarce, but consistently negative. The positive effect of elevated CO2 levels appeared to attenuate with simultaneous exposure to elevated temperatures. Data on impacts of environmental change on nutritional quality of FN&S were sparse, with mixed results.

**Discussion:**

In the absence of adaptation strategies, predicted environmental changes will reduce yields of FN&S. With global intake already well-below WHO recommendations, declining FN&S yields may adversely affect population health. Adaptation strategies and careful agricultural and food system planning will be essential to optimise crop productivity in the context of future environmental changes, thereby supporting and safeguarding sustainable and resilient food systems.

## 1 Introduction

There is now well established evidence that human-driven changes to our planet’s environment are accelerating at a pace that threatens human health through altered functioning of global systems (1). Changes, such as rising carbon dioxide (CO_2_) levels, changing rainfall patterns, deviations in temperature trends and tropospheric ozone (O_3_) depletion, pose a challenge to agricultural yield and nutritional content of foods. If not tackled by adequate adaptations strategies, these changes threaten to impact food security (2). Global research efforts, focussing mainly on staple crops (2–9) and more recently vegetables and legumes (10), have demonstrated reduced crop yield and nutrient quality in response to environmental stressors. However, there has, to-date, been little focus on fruits, nuts and seeds, which are an important source of nutrients and are associated with positive health outcomes.

Fruits are a major source of nutrients and bioactive compounds important for health and disease prevention. In the Global Burden of Disease 2017 models, inadequate intake of fruit is among the top three leading dietary risk factors for deaths and disability-adjusted life-years (11), and modelled estimates have suggested that climate-induced changes to fruit and vegetable consumption would be one of the largest related drivers of climate-related deaths by 2050 (12). A diet low in nuts and seeds is the fourth leading dietary risk factor for non-communicable diseases (NCDs) according to the 2017 Global Burden of Disease study, and insufficient intake of nuts and seeds accounts for over 2% of deaths globally (11). Previous meta-analyses have shown that nut consumption is inversely associated with fatal and non-fatal ischaemic heart disease, diabetes (13), cholesterol and triglycerides (14). Meta-analyses investigating the effect of consumption of seeds on health outcomes are less abundant, although there is some suggestion flaxseed consumption is associated with reduced blood pressure (15). Tree nuts (such as almonds, walnuts and pistachios), groundnuts (such as peanuts) and seeds are energy and nutrient-dense foods, however their consumption is often undervalued by national dietary guidelines (16).

Global healthy and sustainable reference diets now advise low amounts of animal products, based on a growing concern about the impact of animal source food production on environmental change, and encourage increased consumption of plant-based foods such as fruit and vegetables (17). Nuts and seeds as well as legumes can also play a pivotal role in providing a healthier, nutrient-dense and longer shelf-life alternative to animal products as a source of protein and other nutrients. Safeguarding an adequate and stable global supply of fruits, vegetables, nuts and seeds is therefore essential.

Fruits, nuts and seeds, like many other crops, are sensitive to changes in environmental exposures throughout the year. The number of hot days, the overall growing season climate and changes in minimum and maximum daily temperatures all substantially affect fruit development (18). For example, higher than usual temperatures during the dormant phase and low water availability during fruit forming of perennial fruit trees could cause significant damage to fruit yield and nutritional quality (18, 19). Similarly with nuts, winter chill is necessary for successful nut tree cultivation, however changes in global temperature trends threaten to reduce winter chill and compromise yields, particularly in warm climates such as California, China and Australia (20). Prolonged periods of drought have also been associated with low production of groundnuts (21), and are projected to become more frequent in dry sub-tropical regions (2). In 2015, North America, Asia and the Middle East accounted for an estimated 35%, 24% and 15% of the global tree nut production respectively (22), however more frequent extreme weather events such as heat waves, flooding and drought in these regions (2) may impact their future production capacity.

To date there has been no systematic review of the impact of environmental changes on the availability and nutritional quality of fruits, nuts and seeds. We here report the findings of a systematic review of available published studies examining the effect of changes in environmental exposures on yield of fruits, nuts and seeds and the nutritional quality of fruits in field and greenhouse settings. We focus on studies that were conducted in standardized business-as-usual scenarios with no involvement of new technologies or changes in agricultural practices.

## 2 Methods

### 2.1 Search Strategy

This systematic review follows the Preferred Reporting Items for Systematic Reviews and Meta-Analyses (PRISMA) guidelines (23). We performed a systematic search of published literature to identify all peer-reviewed field and greenhouse studies that explored the effect of a single or combination of environmental exposures on yields and/or nutritional quality of the 20 most commonly consumed fruits^1^; and yields of the 15 most commonly consumed nuts^2^ and seeds globally (Appendix). The search for papers on nuts and seeds was performed in July 2018 and covered the papers published between 1 January 1970 and 30 June 2018, whilst the search for papers on fruits was performed in November 2018 and covered papers published between 1 January 1970 and 21 November 2018. Most commonly consumed varieties of each crop group were determined by studying the Food and Agriculture Organisation (FAO) food balance sheets (24). The evaluated environmental exposures were defined as major projected changes over the coming decades identified by the Rockefeller Lancet Commission on Planetary Health (1), namely ambient temperature, water availability, water salinity, elevated tropospheric CO_2_ concentration, and elevated tropospheric O_3_ concentration. Specifically, we considered water salinity either through flooding, saline ground water or saline irrigation water, and did not include papers on soil salinity. The primary outcomes were the percentage change in yield of fruits, nuts or seeds (exposure versus baseline) and nutritional quality of fruit (concentration of nutritionally-relevant substances). All nutritionally-relevant substances reported in included papers were considered, namely: flavonoids, ascorbic acid (vitamin C), carotenoids, phenolic compounds, and antioxidants (including antioxidant activity).

A search of seven databases was carried out in conjunction with a second systematic review evaluating the impact of environmental change on vegetables and legumes (10). Databases searched were OvidSP MEDLINE, OvidSP EMBASE, EBSCO GreenFILE, Web of Science Core Collection and OvidSP AGRIS: to identify papers on fruits two additional databases were searched: Scopus and OvidSP CAB Abstracts. The search strategy was first developed and refined in Web of Science Core, then adapted as necessary for the remaining databases. In addition to database searching, citation lists of included papers were hand-searched for relevant studies, and subject experts were contacted (n=4). Where full-texts were unavailable (n=7), we contacted all authors and one author provided us with one additional paper.

### 2.2 Selection Criteria and Data Extraction

We included experimental studies conducted in field and greenhouse settings, written in English, French, Spanish, German or Dutch; modelling studies were excluded. Titles and abstracts were screened for relevance by four reviewers for fruits papers (PS, FB, CC, PH) and two reviewers for nuts and seeds papers (SN, CC). Full-texts were read by two reviewers (FB, PH, CC or SN), and any discrepancies were discussed and settled with a third reviewer (PS or HT). A single reviewer performed data extraction (PS, FB, PH, HT or SN), of which a random 20% sample was checked by a second reviewer (CC). Extracted data included study location, publication year, study design (field or greenhouse study), environmental exposure considered (including baseline and experimental levels), crop type and group, yields at baseline and under experimental conditions, and nutritional quality parameter concentration at baseline and under experimental conditions.

### 2.3 Study Quality and Risk of Bias

Papers identified for inclusion were assessed for quality using a modified version of the Critical Appraisal Skills Programme (CASP) checklist for randomised controlled trials (25), adapted for relevance to this interdisciplinary review (Supplement B). Parameters relating to randomisation, blinding and cost-effectiveness were excluded from the checklist. Papers were assigned a quality score ranging from 0 to a maximum of 5 relating to the following criteria: 1) clear description of the study design, 2) appropriate comparison group, 3) clear description of the methods, 4) rigorous and clearly described analysis, including critical examination of potential biases, and 5) precision estimate of the measure of effect (confidence intervals and/or standard deviations). Papers not reporting precision estimates were included in the review, however only papers that reported precision estimates of measured effects were to be included in pooled analysis. Papers not meeting a quality score of at least 4 were excluded.

### 2.4 Data Analysis

The absolute differences in outcome between baseline and exposure were used to derive percentage changes in yield or change in concentration of certain nutritional quality parameters for each individual experiment reported by the included studies. Results were grouped by environmental exposure (single or combination) and crop type (nuts and seeds) or crop group (fruits). Fruits were sub-divided into aggregates of similar dietary function. They were defined as: berries (including grapes and strawberries); pome (including apples and pears); cucurbits (including several types of melon); citrus (including oranges and lemons; drupe (including peaches and apricots); and bromeliads (including pineapple). For the purposes of this analysis, field and greenhouse studies were combined due to the experiments having been conducted under a variety of ambient and soil conditions. Sensitivity analysis showed that the direction and scale of findings in the two study designs were similar.

Scatter plots were used to visually display the relationship between changes in outcome and the evaluated range of the environmental exposures. Where measurement units for the exposures differed amongst the included studies, the percentage change in exposures were used. Crude summary estimates, here named “mean yield change”, along with their corresponding 95% confidence intervals (CIs), were calculated where a minimum of three different studies examining the same range of environmental exposure were identified. Due to the clustered nature of the data (i.e. multiple experiments in a single paper), the Huber (sandwich) estimate of variance (26) was used to estimate means, with each paper representing a cluster unit. The impact on nutritional indicators was analysed separately for each crop group and environmental exposure. Pooled analysis was conducted when a minimum number of three papers reported precision estimates for the effect of the same exposure on crop yield or nutritional quality. All plots and statistical analyses were performed in STATA 15.0 (StataCorp, LLC, College Station, Texas, USA).

## 3 Results

### 3.1 Screening

**Figure 1:**
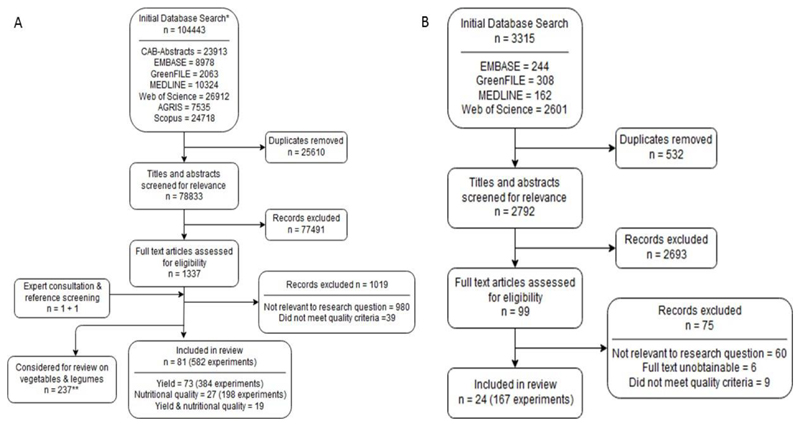
PRISMA chart showing the numbers of papers at each stage of the screening process. A. Fruits; B. Nuts and seeds. *Covering the combined search for systematic reviews on 1)vegetables and legumes – published elsewhere (10) and 2) fruits. **Two papers analysed both fruits and vegetables/legumes.

The initial database search identified 104,443 titles for fruits, and 3,315 titles for nuts and seeds. After removal of duplicates and screening of titles and abstracts, 1,337 potentially relevant papers for fruits (including one paper identified through consulting experts in the field and one paper identified by reference screening), and 99 potentially relevant papers for nuts and seeds remained for assessment of eligibility and quality. Of these, 1,256 papers were excluded during full text screening for fruits, and 75 during nuts and seeds screening. A total of 81 papers (582 experiments) on fruits were included in the final analysis, of which 73 reported on yields and 27 reported on nutritional quality (19 reported on both). In the final analysis on yields of nuts and seeds, 24 papers (167 experiments) were included (Figure 1).

**Figure 2:**
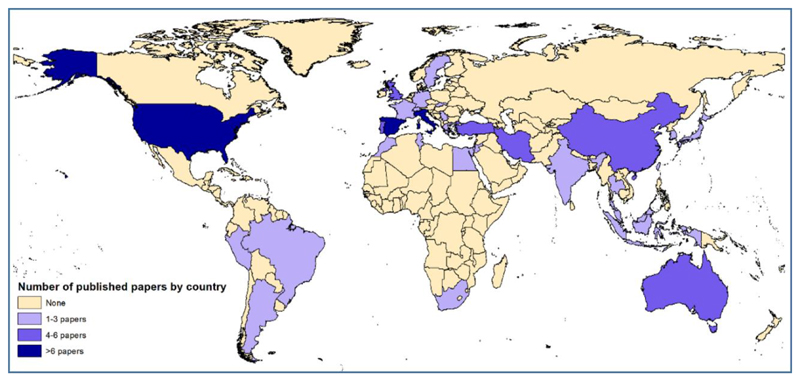
Geographical spread of experiments on fruits, nuts and seeds reported in papers identified for this systematic review

Sixty-five papers on fruits reported on field studies and 16 papers on greenhouse studies (including one study in a rain shelter - Supplement C). Of the 24 included nuts and seeds papers, 15 took place in field settings and 9 within greenhouses or related structures such as growth chambers, glasshouses and rain shelters. Experiments were conducted in 32 different countries, with the highest concentration in Spain (17 papers) and the United States (17 papers – Figure 2).

Berries were the most commonly studied fruit group (34 studies, 204 experiments), and peanuts were the most frequently studied of the nuts and seeds crops (11 papers, 78 experiments) (Table 1). Water availability was the most commonly assessed environmental exposure (348 fruits experiments; 89 nuts and seeds experiments).

### 3.2 Impact of single environmental exposures

#### 3.2.1 Water Availability

**Figure 3:**
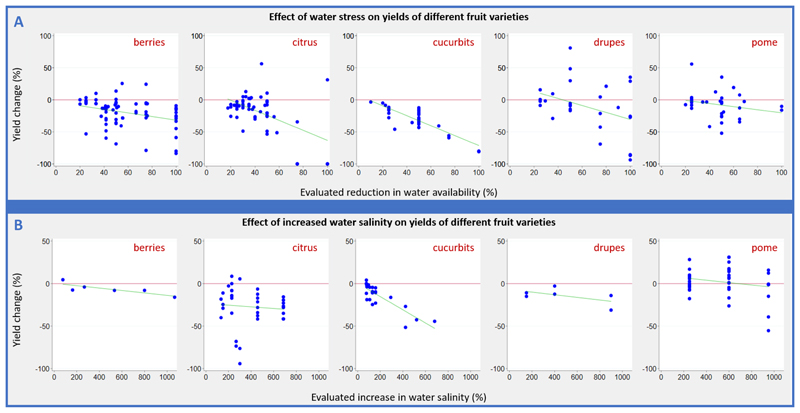
A) Change in fruit yield in response reduced water availability - by crop group & B) Change in fruit yield in response increased water salinity - by crop group

We identified 48 papers (44 field studies, three greenhouse studies, and one outdoor rain shelter study; 348 experiments) that reported on the effect of reduced water availability on fruit yields (Figure 3A). The evaluated reduction in water availability ranged from 10% to 100%. Yield changes in berries resulting from a 50% reduction in water availability (five studies; nine experiments) were negative (range -68.8% to +13.7%; mean yield change -20.7%; 95% CI -43.1% to -1.7%). Negative yield changes resulting from a 50% reduction in water availability were also seen in citrus (four studies; 10 experiments; range -53.5% to +10.6%; mean yield change -19.6%; 95% CI -31.2% to -8.1%), cucurbits (five studies; 18 experiments; range -43.3% to -12.3%; mean yield change -28.0; 95% CI -31.5% to -24.5%), and pome crops (three studies; seven experiments; range -52.1% to +10.4%; mean yield change -24.3%; 95% CI -49.2% to 0.6%). A non-significant positive mean yield change was demonstrated from a 50% reduction in water availability in drupe crops (four studies, eight experiments; range -16.9 to 81.0%; mean yield change 13.1%; 95% CI -26.7 to 53.0%).

**Figure 4:**
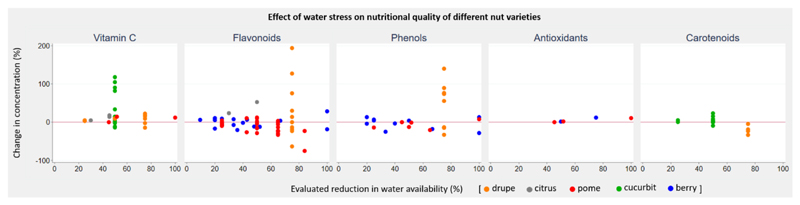
Change in five quality parameters of fruit groups in response to reduced water availability.

**Figure 5:**
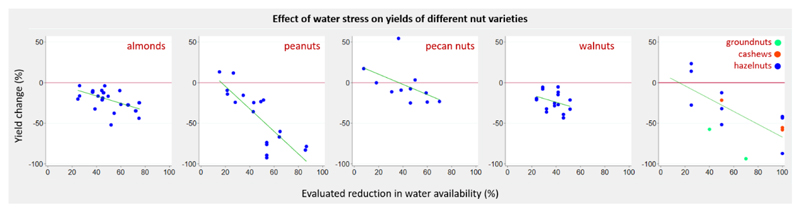
Change in yields of nuts in response to change of water availability - by crop group.

Eighteen studies (17 field studies, one greenhouse study; 131 experiments) reported on the effect of reduced water availability on nutritional quality of fruits. The evaluated reduction in water availability ranged from 9.5% to 100%. Water stress largely resulted in increased nutrient concentrations in citrus and cucurbit crops, and decreased concentrations in pome crops. No consistent dose-response pattern in quality parameters could be observed in response to water stress (Figure 4). Eleven studies (43 experiments) reported the effect of a 45% to 55% reduction in water availability on fruits (all quality parameters and crop groups combined), and mean change in concentration of quality parameter was positive but non-significant (range -28.5% to 117.9%; mean concentration change 12.1%; 95% CI -4.6% to 28.8%). One study reported uncertainty estimates and no pooled analysis was performed. We identified 13 papers (12 field studies and one outdoor rain shelter study; 89 experiments) examining the effect of restricted water availability on nut yields. The evaluated reduction in water availability ranged from 7.7% to 100%. The majority of experiments reported negative yield change across almonds, peanuts, and walnuts, with yields decreasing as water stress increased (Figure 5). Pecan yields were positive at lower levels of water stress; however, as water stress increased beyond 40% yields became negative. No studies reported uncertainty estimates and no pooled analysis was performed.

#### 3.2.2 Water Salinity

We identified 12 papers (11 field studies, one greenhouse study; 112 experiments) assessing the effect of water salinity on fruit yields (Figure 3B). All studies measured salinity in dS/m. The evaluated increase in water salinity ranged from 0.15 to 7.3 dS/m, and was converted to a percentage increase from baseline salinity levels. Yield changes in response to increased water salinity were largely negative across berries, citrus, cucurbits and drupe crops, with yields decreasing as water salinity increased. Seven studies (49 experiments, all crop groups combined) evaluated yield changes in response to a 1 to 2 dS/m increase in water salinity. This resulted in a -4.9% non-significant mean yield change (range -55.3% to 31.0%; 95% CI -14.7% to 4.0%), while a 3 to 4 dS/m increase in water salinity resulted in a -28.2% mean yield change (five studies; 22 experiments; range -94.2% to 5.6%; 95% CI -53.0% to -3.4%). Two studies reported uncertainty estimates; therefore, no pooled analysis was performed.

Two studies (one field and one greenhouse study, eight experiments) assessed the effect of water salinity on three nutritional quality parameters in strawberries and nectarines. Across all experiments, an increase in salinity (ranging from 0.323 to 1 dS/m) resulted in increases in flavonoid, anti-oxidant and phenol concentrations.

Three field studies (22 experiments) assessing the effect of water salinity on peanuts, rapeseed and pistachio yields were identified. The evaluated increase in salinity ranged from 2.0 to 10.1 dS/m. Due to the wide range of exposure changes evaluated and paucity of studies, mean yield changes could not be calculated. However, negative yields were seen in peanuts exposed to salinity levels of 3 dS/m and above, whilst yields of rapeseed and pistachio became negative at levels of salinity above 5 dS/m.

#### 3.2.3 Carbon Dioxide (CO_2_)

We included nine papers (four field studies, five greenhouse studies; 21 experiments) reporting on the impact of changing atmospheric CO_2_ levels; all but two studies reported on berries. The evaluated change in CO_2_ concentrations ranged from +37.0% to +200% and were not all relevant in terms of projected increases in atmospheric CO_2_ over coming decades. Yield changes were largely positive in response to exposure to increasing levels of CO_2_. A positive mean yield change was demonstrated from a 75% to 100% increase in CO_2_ concentration with all crop groups combined (seven studies; 12 experiments; range -23.3% to +133.4%; mean yield change +37.8%; 95% CI +4.1% to +71.5%). Nutritional quality was reported in two papers (one field and one greenhouse study; nine experiments) all reporting on berries. No consistent pattern of change in concentrations of flavonoids and phenols due to increased CO_2_ levels was observed.

**Figure 6:**
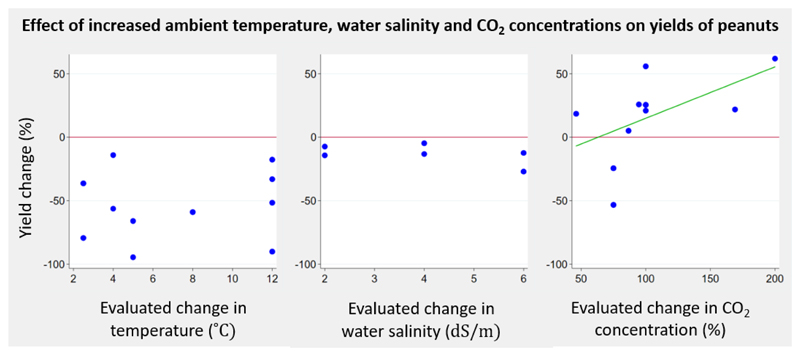
Change in yields of peanuts in response to change of temperature, salinity levels and CO2 concentrations - by crop group

We identified six studies (one field study, five greenhouse studies; 11 experiments) investigating the effect of elevated CO_2_ levels on production of nuts. All papers focused on peanuts. The evaluated increase in CO_2_ levels ranged from +46% to +200%. An increase in peanut yields in response to increasing changes in CO2 levels was observed (Figure 6). A non-significant positive mean yield change was demonstrated from a 75% to 100% increase in CO_2_ concentration (six studies; eight experiments; range -53.3% to +55.9%; mean yield change 10.1%; 95% CI -30.0% to +50.3%). Only two papers reported uncertainty estimates and no pooled analysis was performed.

#### 3.2.4 Temperature

We identified six studies (one field study, five greenhouse studies; 14 experiments) assessing the impact of ambient temperature change on fruit yields. The evaluated increase in temperature ranged from +1°C to +16°C and a variety of baseline temperatures were considered (20°C to 33°C). Considering experiments with a baseline temperature above 25°C, a decrease in berry yields in response to increasing temperatures was observed. Three studies (one field study, two greenhouse studies; 40 experiments) assessed the impact of an increase in temperature on nutritional quality of fruits (berries and pome). Of these, two studies (one study on berries, and one study on pomes) reported a decrease in vitamin C concentrations, but no clear pattern of change in flavonoid concentrations was demonstrated.

We included five studies (all greenhouse studies; 14 experiments) investigating the effect of temperature change on nut yields. All papers focussed on peanuts. The evaluated increase in temperature compared to baseline conditions ranged from +2.5°C to +12°C. Yield changes in response to increasing temperatures were positive in experiments where the baseline temperature was 20°C or below and negative in experiments with higher baseline temperatures (28-33°C) (Figure 6). No study reported uncertainty estimates and no pooled analysis was performed.

#### 3.2.4 Ozone (O_3_)

We identified three studies (all field studies; five experiments) that reported on the impact of O_3_ concentration on fruit yields. Studies evaluated changes in O_3_ concentration ranging from +88% to +143% above baseline levels; in berries and drupe yield changes were negative, while in cucurbits yield changes were positive. None of the identified studies reported on the effect of O_3_ on nutritional quality of fruits.

We included two papers (one field, one open-top chamber; 3 experiments) reporting the impact of O_3_ concentration on production of peanuts and linseed. All experiments resulted in negative yields.

### 3.3 Impact of combined environmental exposures

#### 3.3.1 CO_2_ and temperature

Three greenhouse studies (four experiments) examined the combined impact of a 4°C to 5°C increase in temperature and a 300 to 360ppm increase in CO_2_ concentration on fruit yield (berry and pome). The combined environmental exposures had little impact on yields of fruits (range -7% to +12% yield change). The impact of the same combination of environmental exposures on nutritional quality of fruit (all berries) was assessed by three greenhouse studies (10 experiments), resulting in a non-significant reduction in mean flavonoid concentration, excluding the study reporting CO_2_ in μmol/mol (range -77.4% to -6.5%; mean flavonoid concentration -37.5%; 95% CI -94.4% to +19.5%).

We identified three studies (two growth chamber and one glasshouse study; eight experiments) assessing the combined impact of a 300-350 ppm increase in CO_2_ concentration and 2.5°C to 12°C temperature increase on peanut yield. Baseline temperatures ranged from 28°C to 33°C. The combined environmental exposures resulted largely in a decrease in peanut yields (range -92% to +3% yield change).

#### 3.3.2 Water availability and Salinity

Two field studies (eleven experiments) evaluated the combined effects of reduced water availability and increased water salinity on pineapples and cantaloupe melons. The experiments assessed a broad range of increases in salinity and reductions in water availability (+0.8 to +5.5 dS/m increase in salinity and between 10 and 50% reduction in water availability). All experiments reported negative yield changes.

One field study (18 experiments) investigated the combined effect of salinity and water availability on peanut yield. Reductions in water availability ranged from -51.4 to -26.6%, and salinity increased from 3 to 7 dS/m. These combined environmental exposures were reported to have a negative impact on peanut yield.

#### 3.3.3 CO_2_ and O_3_

One field study (two experiments) assessed the combined impact of elevated CO_2_ and O_3_ on peanut yield. The impacts on yield were inconclusive.

## 4 Discussion

This systematic review summaries the current available experimental evidence of the impact of potential future environmental changes on yields of fruits, nuts and seeds, and nutritional quality of fruits, under a business-as-usual scenario. While some experimental conditions were relatively heterogeneous, several consistent findings emerged. Our results suggest that reduced water availability, increased O_3_ concentrations, elevated temperatures above 28°C and increased water salinity have negative impacts on fruit, nut and seed yields. Positive effects on berry and peanut yields were seen under increased CO_2_ concentrations, however; the positive effect on yields of raised CO_2_ was found to be attenuated by elevated temperatures in experiments with combined environmental exposures.

### 4.1 Comparison with other literature

Our findings relating to nuts and seeds are in line with a number of modelling studies predicting negative cereal yields in response to environmental change, in crops such as rice, maize and wheat (3, 4, 27). A decrease in availability of these staple crops has worrying implications for future food security. Reduced availability of other nutritionally relevant crops such as fruits, vegetables, nuts and legumes would also threaten food security, especially from a dietary (or nutrient) diversity perspective. A recent systematic review on the effect of environmental changes on vegetable and legume yields and nutritional quality found that under a business-as-usual scenario, environmental changes are likely to have substantial negative impact on yields (10), in keeping with our findings presented here regarding fruit, nuts and seeds.

Precipitation is predicted to decrease in many arid sub-tropical areas (2) where many crops such as peanuts, almonds, citrus and drupe fruits are often grown. Furthermore, reduced precipitation could increase water extraction for irrigation, which – in turn – can lead to over-exploitation of aquifers and subsequent freshwater declines. An adequate supply of water is necessary for plant growth and hence crop yield, and water stress can affect normal growth processes such as cell expansion and regulation of photosynthesis (28). However, water stress can affect different crops in different ways, for example the growth phase of hazelnuts (29) and the reproductive phase of peanuts (30) are particularly sensitive to water stress, whereas almonds are relatively drought resistant but do respond to severe water deficits during the stress-sensitive vegetative growth and post-harvest phases (31). Similarly with pecans, the timing of applied water stress influences maximum nut production (32). Whilst our review demonstrated a largely negative impact on reduced water availability on fruit and nuts, these variations in water requirements and periods of water stress sensitivity may help explain the heterogeneity in results between the included papers. While this review focussed on the effect of reduced water availability, a particular issue for dry sub-tropical regions, some varieties of fruit, nuts and seeds are grown in wet tropical areas, and others such as walnuts, hazelnuts, pomes and berries thrive in more temperate climates. Whilst predictions of reduced rainfall are less profound in these regions, changing precipitation patterns may lead to flooding, particularly in tropical areas, with likely implications of reduced crop yields (1).

The findings of potential negative impacts on fruit, nut and seed yields resulting from increased salinity and increased temperature are in line with our current understanding of the impact of salinity and temperature on staple crops (33, 34). The salt tolerance of many vegetables is also low, with decline in yields shown at salinity levels above 4 dS/m (35). Increased salinization is detrimental to plant growth, size and productivity (36). Although outside the scope of this review, saline water intrusion often has a substantial impact on soil salinity. One study in Bangladesh has estimated that increased saltwater intrusion due to effects of environmental change will result in a 39% increase in soil salinity in coastal regions by 2050 (37). It has been estimated that plants grown in saline soils, characterised by an electrical conductivity of 4 ds/m or over (38), undergo osmotic stress and root growth disturbances, often accompanied by impaired nutrient uptake as a result of ion imbalances, leading to decreased yields (35). However, further salinity studies on a wider variety of fruit, nut and seed crops are required in combination with other environmental exposures, in particular water availability in arid regions, in order to fully understand the impact of projected environmental changes on yields. As demonstrated with peanuts, increasing severity of water restriction augmented the effect of salinity, although previous studies on amaranth suggest the effect of these two stressors is not additive (39).

Our results suggest that the sensitivity of peanuts to increased temperature depends upon the baseline temperature. Different stages of peanut growth require different temperatures: vegetative growth is optimal between 25°C to 30 °C (40). This may explain why a 4°C rise in temperature lead to a decrease in yields in experiments with a baseline temperature of 28°C or greater only; a 4°C rise in temperature at a baseline of 20°C would provide near optimal growing conditions. Peanuts are typically grown in tropical and subtropical regions (41), where seasonal temperature extremes are predicted to exceed any extreme temperatures recorded to date as a result of climate change-induced global temperature increases (1). Therefore, without adaptation strategies, the predicted increase in mean global temperature poses a threat to agricultural production of peanuts.

We identified a potentially beneficial effect of CO_2_ on berry and peanut yields, in keeping with a number of other studies evaluating the effect of CO_2_ on yields of rice (42), potatoes (43), peppers (44), and lettuce (45), amongst other crops. This effect is thought to be due to stimulation of photosynthesis by CO_2_ in C_3_ crops (inclusive of peanuts, rice, wheat and many fruits and vegetables), which enhances productivity (46). However, in contrast to the positive effect of increased CO_2_ on yields, a detrimental effect on nutritional quality has previously been found: elevated CO_2_ reduced concentrations of iron and zinc in C_3_ grains and legumes (6). Additionally, our review found some evidence that the beneficial effect of CO_2_ on yields was attenuated by simultaneous exposure to increasing temperature. It has previously been suggested that certain climate change exposures, i.e. increased temperature and water stress, that have negative impacts on yields, may be attenuated by the positive yield impacts of increased atmospheric CO_2_ (47). This was supported in a previous Temperature by Free-Air CO_2_ Enrichment (T-FACE) experiment on soybeans, in which the effect of a combined 200 ppm increase in CO_2_ and 3.5°C elevation in temperature negated the negative effect of increased temperature alone; but also the positive effect of elevated CO_2_ alone (48). Similarly no synergistic effects of temperature and CO_2_ were shown with rice (42), and beans, amongst other crops. As the continued changes to the planet’s environment are likely to encompass both elevated CO_2_ and temperature, it is arguably of more practical relevance to consider environmental exposures in combination.

A further effect of environmental change on the planet’s ecological systems is a global decline in pollinator populations that are essential for promoting yields and nutritional quality of many crops (49), including nutritionally relevant nuts, seeds, fruits and vegetables (50). Modelling of pollinator decline scenarios suggests that complete pollinator loss would result in a 22% reduction in global supply of fruits, nuts and seeds, contributing to a significant increase in NCDs and micronutrient deficiencies - in particular Vitamin A deficiency (50). We did not identify experimental studies investigating the effect of pollinator loss on yields of fruit, nuts and seeds using the search terms in this review, highlighting a gap in the literature relating to an important threat to the global food supply.

The health benefits of consuming not only (starchy) staples but also a wide variety of fruits, vegetables, legumes, nuts and seeds are now widely recognised, in prevention of both micronutrient deficiencies and non-communicable diseases (51). Maintaining adequate production, availability and nutritional quality of these crops is thus required to ensure good quality and quantity of produce to meet the health needs of the current and growing future populations.

### 4.2 Strength and limitations

To our knowledge, this is the first systematic review assessing the impact of environmental changes on yields of nuts and seeds. We performed a thorough and systematic search of published literature to identify all relevant papers, and methodological and reporting quality of all eligible papers was assessed to minimise sources of bias in our synthesised summary of the evidence.

There are however a number of limitations to our review. Firstly, only 17 of the 81 included papers (21%) provided uncertainty estimates for the outcome. As these were for different environmental exposures, our ability to perform quantitative analyses was limited. Secondly, the range of fruit groups assessed was limited for some environmental exposures; the overall nuts and seed varieties assessed was limited to eight nut and two seed types, with paucity of data for crops other than peanuts, and therefore our review does not provide a complete picture of the effect of environmental change on the diverse range of fruits, nuts and seeds. Lastly, we did not account for differences in application of the environmental stressors under experimental conditions; studies used different strategies to “mimic” drought, ranging from substantial but stable reduction in watering during all phenological stages of fruit growth, to specific intermittent water cuts. For example, the extent to which peanut yields are affected by decreased water availability depends on factors such as duration, intensity, and the timing of water stress (52). Although we were able to account for intensity, the differences in timing and duration of water stress between studies were not accounted for, but may have influenced differences in our comparison of the studies.

There are additional limitations to consider arising from the heterogeneity of methodologies used by the included papers. Firstly, yield measurements were inconsistent across the papers, for example some reported seed yield in tonnes/hectare or grams/nut/meter^2^, whilst others reported only yield components such as pod biomass or seed weight in grams. The effects of environmental stressors can affect plant growth at different stages, and therefore mediate their effect on different yield components such as seed or pod size, weight, branch number, plant biomass and total dry weight to differing extents (30, 53). Whilst we were unable to directly compare the absolute effect on yields, the change in percentage yields or yield components were calculated in order to facilitate some comparison between studies. Secondly, there was some variation in the methodology of measuring environmental exposures within the included papers. For example, four different nomenclatures were used in reporting water availability. Thirdly, many of the included studies were conducted with the primary aim of investigating mechanisms to increase yields and/or quality or to explore exposure-resistant varieties, therefore the levels of change in environmental exposures were not always a true reflection of likely future environmental change scenarios. For example, fruit cultivars under investigation may have been more resilient than the “average” cultivar, therefore not demonstrating the full picture of the impact of environmental stressors on yield or quality.

Lastly, issues in style of reporting resulted in limited possibility for data extraction, which led to exclusion of several papers.

### 4.3 Implications and Policy Relevance

The agricultural sector now faces the challenge of producing enough nutritious food in a changing environment, while minimising the environmental footprint of food production. In addition to food security, livelihoods and health are likely to be affected, should the environment continue to change along current trajectories. Global consumption of fruits was under half the recommended intake level of 250g/day in 2017, and nuts and seeds consumption was well under a quarter of the recommended optimal intake level of 21g per day; the current mean global consumption is estimated at approximately 100g/day for fruits, and 3g/day for nuts and seeds (11). Reduction in fruit, nut and seed yields is likely only to widen that gap, contribute to an increased risk of non-communicable disease and micronutrient deficiencies, while also impeding efforts to shift towards more sustainable food systems due to decreasing availability of healthy and nutritious alternatives to animal-sourced foods.

The vast majority of global fruit production is based in tropical and sub-tropical parts of the world (24) that are expected to be disproportionately affected by changing environmental exposure levels (2). Tropical and sub-tropical fruits are consumed both in the country of origin as well as temperate countries; substantial reductions in yields may therefore affect global markets and challenge global availability to a greater extent than other food groups for which local and regional trade and more prominent with a wider range of production zones. Several indirect economic impacts may also arise, especially within the producing nations. For example, raised tropospheric O_3_ concentration increases visible bruising of fruits which reduces market value (54), and this can result in agricultural revenue loss. Reduced labour productivity and exhaustion due to heat stress may also compound its direct effect on fruit yields (55). Most susceptible in this case are often those in the lowest income brackets who commonly perform the majority of agricultural production activities manually.

What will become increasingly important in efforts to ensure the resilience of fruits, nuts and seeds in our diets is a focus on sustainable production; as certain nut species are highly water demanding and relatively vulnerable to water stress, dietary shifts may be necessary towards the less water intensive nut types. Although the shift away from animal source foods towards more planted-based sources is estimated to substantially reduce greenhouse gas emissions (17), water use may well be higher if consumption of certain animal products are substituted by water intensive alternatives. For example, almond milk has a substantially higher water use than dairy milk (56). It may therefore be useful to re-think sustainability-based dietary recommendations with consideration of within-group food aggregation.

In order to respond to changing environmental conditions and maintain the supply of nutritionally important crops, adaptation strategies will be required, such as cultivating resilient crop varieties, efficient irrigation systems, novel pollination techniques and agricultural innovations. It is likely to be the poorest economies and least climate resilient countries who will be most affected by environmental change, but as this will indirectly affect supply of crops to other regions, a global multi-sector response with development and implementation of locally-relevant strategies will be essential.

### 4.4 Future Research

Our study highlights two important gaps in the current evidence-base around the impact of environmental change on yields and nutritional quality of food crops, that could be addressed in future research. First, development of further standardisation and reporting guidelines for agricultural (or wider planetary health) studies, particularly concerning estimate uncertainties, would increase the validity and reliability of future evidence synthesis efforts in this area. Secondly, parameterization of projection models for yields and nutritional quality of fruits, nuts and seeds (as well as vegetables and legumes) under different environmental change scenarios will require detailed information on a large amount of different environmental exposure and crop impact combinations. In contrast, focussing on an evidence synthesis around the physiological drivers and mechanisms through which these environmental exposures affect certain fruits, vegetables, legumes, nuts and seeds, might allow construction of crop aggregates that could reduce the complexity of such models and enable robust yield and nutritional quality projections of nutritionally important crops globally.

### 4.5 Conclusion

Our review identified a number of papers assessing the impact of environmental stressors on the yield of a small range of fruits, nuts and seeds. Our findings suggest that under a business as usual scenario, yields of fruits, nuts and seeds are likely to decrease in response to environmental change. Given the importance of fruits, nuts and seeds to health, and contribution to adequate micronutrient and calorie intake, this will likely have negative implications for food security, nutrition, and NCD risk – especially in food insecure areas. Despite the inherent limitations of performing a systematic review in this field, these novel findings are of importance for research and policy in agricultural development, food security, and global public health. Our review highlights the need for further research using standardised methodologies, including reporting of uncertainty estimates, to assess environmental impacts on a more diverse range of nutritionally relevant crops, in order to fully understand the risk to dietary diversity and nutrition. Additionally, our review contributes to a growing number of inter-disciplinary systematic reviews bringing together the health, environmental and food systems sectors, further demonstrating the benefit of working across related fields to provide evidence for the urgent need to find solutions to improve the health of people and our planet.

## Supplementary Material

Supplement A,B and C

## Figures and Tables

**Table 1:** Number of experiments carried out for each crop, by type of environmental exposure – combining experiments measuring impact on yields and experiments measuring impact on nutritional quality of A) Fruits and B) Nuts and Seeds. (Shading by quintiles)

Exposure	Number of experiments											
	A. Fruits							B. Nuts and Seeds				
	Berries	Cucurbits	Citrus	Drupe	Pome	Bromeliads	Total	Peanuts	Almonds	Other nuts ^a^	Seeds ^b^	Total
Increased CO_2_ concentration	27	2	0	0	1	0	**30**	11	0	0	0	**11**
Increased O_3_ concentration	1	2	0	2	0	0	**5**	1	0	0	2	**3**
Increased temperature	52	0	0	0	2	0	**54**	14	0	0	0	**14**
Reduced water availability	99	53	60	61	75	0	**348**	18	30	41	0	**89**
Increased water salinity	12	24	37	6	41	0	**120**	6	0	12	4	**22**
Increased CO_2_ concentration & increased temperature	13	0	0	0	1	0	**14**	8	0	0	0	**8**
Reduced water availability & increased salinity	0	9	0	0	0	2	**11**	18	0	0	0	**18**
Increased CO_2_ concentration & increased O_3_ concentration	0	0	0	0	0	0	**0**	2	0	0	0	**2**
**Total**	**204**	**90**	**97**	**69**	**120**	**2**	**582**	**78**	**30**	**53**	**6**	**167**

a Bambara groundnut, cashew, hazelnut, pecan, pistachio and walnuts

b Linseed and rapeseed.

## Footnotes

1 For the purposes of this review, fruit crops such as tomatoes, cucumbers, peppers, avocados, courgettes, pumpkins and aubergines that are typically consumed as vegetables were excluded.

2 Including legumes commonly consumed as nuts
