# Supplementary material for: The impact of environmental changes on the yield and nutritional quality of fruits, nuts and seeds: a systematic review
Source: Environ Res Lett. Author manuscript; Available in PMC 2020 Feb 4. (PMC7000241; doi:10.1088/1748-9326/ab5cc0)
Supplement: Supplementary file 1 [file EMS85315-supplement-Supplement_A_B_and_C.pdf]

Supplementary Information

The impact of environmental changes on the yield and nutritional quality of fruits, nuts and seeds: a systematic review

A. Database Search Strategies.

i. Fruits Yields

AGRIS

1. ((climate adj1 (change* OR variability OR disruption)) OR (land* adj1 degradation) OR ((increas* OR elevat* OR fertili#ation) adj2 (carbon-dioxide OR carbondioxide OR "carbon dioxide" OR co2)) OR (soil adj2 (fertili#ation OR eutrophication)) OR “global warming” OR “greenhouse effect*” OR ((water OR soil*) adj4 (sodium OR salini#ation OR saline OR salinity)) OR global temperature* OR (temperature* adj2 (increase* OR variab* OR trend*)) OR “extreme weather” OR “extreme temperature” OR (heatwave* OR heat-wave* OR "heat wave") OR (cold adj1 extreme*) OR (rise adj4 (sea level OR sea-level OR sealevel)) OR flood* OR “storm surge” OR landslide* OR (wildfire* OR wild-fire* OR wild fire) OR windstorm* OR cyclone* OR (humidit* adj2 (increas* OR change* OR elevation* OR variabilit* OR pattern*)) OR “rainfall pattern*” OR ((acid OR acidifi*) adj1 rain*) OR “carbon footprint” OR (ozone* adj2 depletion) OR “climate pollutant*” OR “extended drought*” OR “cloud cover” OR (change adj2 ("land use" OR "land cover")) OR “soil erosion” OR “soil quality” OR (nutrient* adj1 (run-off OR runoff OR "run off")) OR ((quality OR availability) adj1 (groundwater OR ground-water OR "ground water" OR freshwater OR fresh-water OR "fresh water" OR surfacewater OR surface-water OR "surface water" OR "irrigation water")) OR aquifer* OR (water adj2 (availab* OR security OR stress OR scarcity)) OR (water adj2 quality) OR biodivers* OR “biological divers*” OR pollinator* OR ((bee OR bees OR insect OR insects) adj2 decline) OR “ecosystem* change*” OR "health of the planet" OR “environment* change*” OR (chemical adj1 contamination) OR (pest* adj4 (crop* OR plant*)) OR (plant* adj1 (pathogen* OR disease*))).ti,ab.

2. (fruit OR watermelon OR banana OR apple OR grape OR orange OR tangerine OR mandarin OR satsuma OR clementine OR citrus OR coconut OR mango OR mangosteen OR guava OR melon OR pear OR pineapple OR vegetable OR bean OR tomato OR onion OR cabbage OR cucumber OR gherkin OR eggplant OR aubergine OR carrot OR turnip OR pepper OR lettuce OR garlic OR brassica OR allium).ti,ab.

3. ((yield adj2 change) OR "crop production" OR "crop growth" OR producti* OR growth OR yield OR harvest OR biomass OR “dry weight” OR “fresh weight”).ti.ab.

CAB Abstracts

1. ((climate adj1 (change* OR variability OR disruption OR warming)) OR (global adj1 (warming OR temperature)) OR “greenhouse effect” OR “elevated CO2” OR ((increas* OR elevat* OR fertili?ation) adj2 (carbon-dioxide OR carbondioxide OR “carbon dioxide” OR CO2)) OR “carbon footprint” OR (ozone* adj2 depletion) OR climate pollutant* OR (soil adj2 (fertili?ation OR eutrophication)) OR ((soil OR water) adj4 (sodium OR salini?ation OR salin*)) OR (temperature adj2 (increas* OR variability OR trend*)) OR extreme weather OR extreme temperature OR heatwave OR

heat-wave OR (cold adj1 extreme) OR ((“sea level” OR sea-level OR sealevel) adj4 rise) OR flood* OR “storm surge” OR landslide* OR wildfire* OR wild-fire* OR tropical cyclone* OR windstorm* OR (humidity adj2 (increase OR change OR elevat* OR variability OR pattern)) OR “rainfall pattern” OR “acid rain” OR “drought stress” OR “water stress” OR “water scarcity” OR “extended drought” OR “cloud cover” OR (change adj1 (“land use” OR “land-use” OR “land cover”)) OR soil erosion OR soil quality OR “nutrient run-off” OR ((quality OR availability) adj1 (groundwater OR ground-water OR “ground water” OR freshwater OR fresh-water OR “fresh water”)) OR aquifer* OR “water security” OR (water adj2 availab*) OR (water adj2 quality) OR biodiverse* OR biological divers* OR pollinat* OR ((bee* OR insect) adj2 decline) OR “ecosystem change” OR “health of the planet” OR planet* health OR environment* change OR (chemical N/1 contamination) OR (pest* adj4 (crop OR plant)) OR (plant adj1 (pathogen* OR disease*)).ti,ab.

2. (fruit* OR watermelon* OR banana* OR apple* OR grape* OR orange* OR tangerine* OR mandarin* OR satsuma* OR clementine* OR citrus OR coconut* OR mango** OR mangosteen* OR guava* OR melon* OR pear* OR pineapple* OR vegetable* OR bean* OR tomato** OR onion* OR cabbage* OR cucumber* OR gherkin* OR eggplant* OR aubergine* OR carrot* OR turnip* OR pepper* OR lettuce OR garlic* OR brassica* OR allium).ti,ab.

3. ((yield adj3 change) OR "crop production" OR "crop growth" OR producti* OR growth OR yield* OR harvest OR biomass OR “dry weight” OR “fresh weight”).ti,ab.

GreenFILE

1. (climate N1 (change* or variability or disruption)) OR (land* N1 degradation) OR ((increas* OR elevat* OR fertilisation OR fertilization) N2 (carbon-dioxide OR carbondioxide OR "carbon dioxide" OR co2)) OR (soil N2 (fertilization OR fertilisation OR eutrophication)) OR global warming OR greenhouse effect OR ((water OR soil*) N4 (sodium OR salinisation OR salinization OR saline OR salinity)) OR global temperature* OR temperature* N2 (increase* OR variab* OR trend*) OR extreme weather OR extreme temperature* OR heatwave* OR heat-wave* OR "heat wave" OR (cold N1 extreme*) OR (rise N4 (sea level OR sea-level OR sealevel)) OR flood* OR storm surge* OR landslide* OR (wildfire* or wild-fire* or wild fire) OR windstorm* OR cyclone* OR (humidit* N2 (increas* or change* or elevation* or variabilit* or pattern*)) OR rainfall pattern* OR ((acid or acidifi*) N1 rain*) OR carbon footprint OR (ozone* N2 depletion) OR climate pollutant OR extended drought OR cloud cover OR (change N2 (land use OR land cover)) OR soil erosion OR soil quality OR (nutrient* N1 (run-off OR runoff OR "run off")) OR ((quality OR availability) N1 (groundwater OR ground-water OR ground water OR freshwater OR fresh-water OR fresh water OR surfacewater OR surface-water OR surface water OR irrigation water)) OR aquifer* OR (water N2 (availab* OR security OR stress OR scarcity)) OR (water N2 quality) OR biodivers* OR biological divers* OR pollinator* OR ((bee OR bees OR insect OR insects) N2 decline) OR ecosystem* change* OR (health* N2 planet) OR environment* change* OR (chemical N1 contamination) OR (pest* N4 (crop* OR plant*)) OR (plant* N1 (pathogen* or disease*))

2. pulses OR banana* OR tomato* OR orange* OR mandarin* OR apple* OR grapes OR plantain* OR Coconut* OR Pineapple* OR Lemon* OR Lime* OR citrus OR Grapefruit* OR Dates OR Olive* OR berries OR strawberr* OR raspberr* OR blackberr* OR melon* OR Cherry OR cherries OR Kiwi* OR plum* OR Pomegranate OR Peach* OR avocado* OR papaya* OR pumpkin* OR onion* OR bean* OR Peas OR Pepper* OR piment* OR Spinach OR Lettuce OR kale OR garlic OR broccoli OR sprout*

3. (yield N3 change) OR "crop production" OR "crop growth" OR producti* OR growth OR yield* OR harvest OR biomass OR “dry weight” OR “fresh weight”

Web of Science

1. TS = (climate near/2 (change* OR variability OR disruption)) OR (land* near/1 degradation) OR ((increas* OR elevat* OR fertili?ation) near/2 (carbon-dioxide OR carbondioxide OR "carbon dioxide" OR co2)) OR (soil near/2 (fertili?ation OR eutrophication)) OR "global warming" OR "greenhouse effect*" OR ((water or soil*) near/4 (sodium or salini?ation or saline or salinity)) OR "global temperature*" OR (temperature* near/2 (increase* OR variab* OR trend*)) OR "extreme weather" OR "extreme temperature*" OR (heatwave* OR heat-wave* OR "heat wave") OR (cold near/1 extreme*) OR ((rise* OR increase*) near/4 ("sea level" OR sea-level OR sealevel)) OR flood* OR "storm surge*" OR landslide* OR (wildfire* OR wild-fire* OR "wild fire*") OR windstorm* OR cyclone* OR (humidit* near/2 (increas* OR change* OR elevation* OR variabilit* OR pattern*)) OR rainfall pattern* OR ((acid OR acidifi*) near/1 rain*) OR "carbon footprint" OR (ozone* near/2 depletion) OR "climate pollutant*" OR "extended drought*" OR "cloud cover" OR (change near/2 ("land use" OR "land cover")) OR "soil erosion" OR "soil quality" OR (nutrient* near/1 (run-off OR runoff OR "run off")) OR ((quality OR availability) near/1 (groundwater OR ground-water OR "ground water" OR freshwater OR fresh-water OR "fresh water" OR surfacewater OR surface-water OR "surface water" OR "irrigation water")) OR aquifer* OR (water near/2 (availab* OR security OR stress OR scarcity)) OR (water near/2 quality) OR biodivers* OR "biological divers*" OR pollinator* OR ((bee OR bees OR insect OR insects) near/2 decline) OR (ecosystem* near/2 change*) OR (health* near/2 planet) OR (environment* near/2 change*) OR (chemical* near/1 contamination) OR (pest* near/4 (crop* OR plant*)) OR (plant* near/1 (pathogen* OR disease*))

2. TS = (pulses OR banana* OR tomato* OR orange* OR mandarin* OR apple* OR grapes OR plantain* OR Coconut* OR Pineapple* OR Lemon* OR Lime* OR citrus OR Grapefruit* OR Dates OR Olive* OR berries OR strawberr* OR raspberr* OR blackberr* OR melon* OR Cherry OR cherries OR Kiwi* OR plum* OR Pomegranate OR Peach* OR avocado* OR papaya* OR pumpkin* OR onion* OR bean* OR Peas OR Pepper* OR piment* OR Spinach OR Lettuce OR kale OR garlic OR broccoli OR sprout*)

3. TS = ("change in yield" OR "yield change") OR ("crop production" OR "crop growth") OR (productivity OR production) OR (growth OR yield) OR (harvest OR biomass) OR ("dry weight" OR dry-weight OR "fresh weight" OR fresh-weight)

Scopus

1. TITLE-ABS-KEY ((climate W/2 (change* OR variability OR disruption)) OR (land* W/1 degradation) OR ((increase* OR elevat* OR fertili?ation) W/2 (carbon-dioxide OR carbondioxide OR "carbon dioxide" OR co2)) OR (soil W/2 (fertili?ation OR eutrophication)) OR "global warming" OR "greenhouse effect*" OR ((water OR soil*) W/4 (sodium OR salini?ation OR saline OR salinity)) OR "global temperature*" OR (temperature* W/2 (increas* OR variab* OR trend*)) OR "extreme weather" OR "extreme temperature*" OR (heatwave* OR heat-wave* OR "heat wave") OR (cold W/1 extreme*) OR ((rise OR increase) W/4 ("sea level" OR "sea-level" OR sealevel)) OR flood* OR "storm surge*" OR landslide* OR (wildfire* OR wild-fire* OR "wild fire*") OR windstorm* OR cyclone* OR (humidit* W/2 (increas* OR change* OR elevation* OR variabilit* OR pattern*)) OR "rainfall pattern*" OR ((acid OR acidifi*) W/1 rain*) OR "carbon footprint" OR (ozone* W/2 depletion) OR "climate pollutant*" OR "extended drought*" OR "cloud cover" OR (change W/2 ("land use" OR "land cover")) OR "soil erosion" OR "soil quality" OR (nutrient* W/1 ("run-off" OR runoff OR "run off")) OR ((quality OR availability) W/1 (groundwater OR ground-water OR "ground water" OR freshwater OR fresh-water OR "fresh water" OR surfacewater OR surface-water OR "surface water" OR "irrigation water")) OR aquifer* OR (water W/2 (availab* OR security OR stress OR scarcity)) OR (water W/2 quality) OR biodivers* OR "biological divers*" OR pollinator* OR ((bee OR bees OR insect OR insects) W/2 decline) OR (ecosystem* W/2 change*) OR

“health of the planet” OR (environment* W/2 change*) OR (chemical* W/1 contamination) OR (pest* W/4 (crop* OR plant*)) OR (plant* W/1 (pathogen* OR disease*)))

2. TITLE-ABS-KEY (pulses OR banana* OR tomato* OR orange* OR mandarin* OR apple* OR grapes OR plantain* OR coconut* OR pineapple* OR lemon* OR lime* OR citrus OR grapefruit* OR dates OR olive* OR berries OR strawberr* OR raspberr* OR blackberr* OR melon* OR cherry OR cherries OR kiwi* OR plum* OR pomegranate OR peach* OR avocado* OR papaya* OR pumpkin* OR onion* OR bean* OR peas OR pepper* OR piment* OR spinach OR lettuce OR kale OR garlic OR broccoli OR sprout*)

3. TITLE-ABS-KEY ((yield W/3 change) OR "crop production" OR "crop growth" OR producti* OR growth OR yield OR harvest OR biomass OR “dry weight” OR “fresh weight”)

EMBASE

1. ((climate adj1 (change* OR variability OR disruption)) OR (land* adj1 degradation) OR ((increas* OR elevat* OR fertili#ation) adj2 (carbon-dioxide OR carbondioxide OR "carbon dioxide" OR co2)) OR (soil adj2 (fertili#ation OR eutrophication)) OR global warming OR greenhouse effect* OR ((water OR soil*) adj4 (sodium OR salini#ation OR saline OR salinity)) OR global temperature* OR (temperature* adj2 (increase* OR variab* OR trend*)) OR extreme weather OR extreme temperature OR (heatwave* OR heat-wave* OR "heat wave") OR (cold adj1 extreme*) OR (rise adj4 (sea level OR sea-level OR sealevel)) OR flood* OR storm surge* OR landslide* OR (wildfire* OR wild-fire* OR wild fire) OR windstorm* OR cyclone* OR (humidit* adj2 (increas* OR change* OR elevation* OR variabilit* OR pattern*)) OR rainfall pattern* OR ((acid OR acidifi*) adj1 rain*) OR carbon footprint OR (ozone* adj2 depletion) OR climate pollutant* OR extended drought* OR cloud cover OR (change adj2 ("land use" or "land cover")) OR soil erosion OR soil quality OR (nutrient* adj1 (run-off or runoff or "run off")) OR ((quality OR availability) adj1 (groundwater OR ground-water OR "ground water" OR freshwater OR fresh-water OR "fresh water" OR surfacewater OR surface-water OR "surface water" OR "irrigation water")) OR aquifer* OR (water adj2 (availab* OR security OR stress OR scarcity)) OR (water adj2 quality) OR biodivers* OR biological divers* OR pollinator* OR ((bee OR bees OR insect OR insects) adj2 decline) OR ecosystem* change* OR "health of the planet" OR environment* change* OR (chemical adj1 contamination) OR (pest* adj4 (crop* OR plant*)) OR (plant* adj1 (pathogen* OR disease*))).ti,ab.

2. (pulses OR banana* OR tomato* OR orange* OR mandarin* OR apple* OR grapes OR plantain* OR Coconut* OR Pineapple* OR Lemon* OR Lime* OR citrus OR Grapefruit* OR Dates OR Olive* OR berries OR strawberr* OR raspberr* OR blackberr* OR melon* OR Cherry OR cherries OR Kiwi* OR plum* OR Pomegranate OR Peach* OR avocado* OR papaya* OR pumpkin* OR onion* OR bean* OR Peas OR Pepper* OR piment* OR Spinach OR Lettuce OR kale OR garlic OR broccoli OR sprout*).ti,ab.

3. ((yield adj3 change) OR "crop production" OR "crop growth" OR producti* OR growth OR yield OR harvest OR biomass OR "dry weight" OR "fresh weight").ti,ab.

MEDLINE

1. ((climate adj1 (change* OR variability OR disruption)) OR (land* adj1 degradation) OR ((increas* OR elevat* OR fertili#ation) adj2 (carbon-dioxide OR carbondioxide OR "carbon dioxide" OR co2)) OR (soil adj2 (fertili#ation OR eutrophication)) OR global warming OR greenhouse effect* OR ((water OR soil*) adj4 (sodium OR salini#ation OR saline OR salinity)) OR global temperature* OR (temperature* adj2 (increase* OR variab* OR trend*)) OR extreme weather OR extreme temperature OR (heatwave* OR heat-wave* OR "heat wave") OR (cold adj1 extreme*) OR (rise adj4 (sea level OR sea-level OR sealevel)) OR flood* OR storm surge* OR landslide* OR (wildfire* OR wild-fire* OR wild fire) OR windstorm* OR cyclone* OR (humidit* adj2 (increas* OR change* OR elevation*

OR variabilit* OR pattern*)) OR rainfall pattern* OR ((acid OR acidifi*) adj1 rain*) OR carbon footprint OR (ozone* adj2 depletion) OR climate pollutant* OR extended drought* OR cloud cover OR (change adj2 ("land use" or "land cover")) OR soil erosion OR soil quality OR (nutrient* adj1 (run-off or runoff or "run off")) OR ((quality OR availability) adj1 (groundwater OR ground-water OR "ground water" OR freshwater OR fresh-water OR "fresh water" OR surfacewater OR surface-water OR "surface water" OR "irrigation water")) OR aquifer* OR (water adj2 (availab* OR security OR stress OR scarcity)) OR (water adj2 quality) OR biodivers* OR biological divers* OR pollinator* OR ((bee OR bees OR insect OR insects) adj2 decline) OR ecosystem* change* OR "health of the planet" OR environment* change* OR (chemical adj1 contamination) OR (pest* adj4 (crop* OR plant*)) OR (plant* adj1 (pathogen* OR disease*))).ti,ab.

2. (pulses OR banana* OR tomato* OR orange* OR mandarin* OR apple* OR grapes OR plantain* OR Coconut* OR Pineapple* OR Lemon* OR Lime* OR citrus OR Grapefruit* OR Dates OR Olive* OR berries OR strawberr* OR raspberr* OR blackberr* OR melon* OR Cherry OR cherries OR Kiwi* OR plum* OR Pomegranate OR Peach* OR avocado* OR papaya* OR pumpkin* OR onion* OR bean* OR Peas OR Pepper* OR piment* OR Spinach OR Lettuce OR kale OR garlic OR broccoli OR sprout*).ti,ab.

3. ((yield adj3 change) OR "crop production" or "crop growth" OR producti* OR growth OR yield OR harvest OR biomass OR "dry weight" OR "fresh weight").ti,ab.

ii. Fruits Nutritional Quality

AGRIS

1. ((climate adj1 (change* OR variability OR disruption)) OR (land* adj1 degradation) OR ((increas* OR elevat* OR fertili#ation) adj2 (carbon-dioxide OR carbondioxide OR "carbon dioxide" OR co2)) OR (soil adj2 (fertili#ation OR eutrophication)) OR “global warming” OR “greenhouse effect*” OR ((water OR soil*) adj4 (sodium OR salini#ation OR saline OR salinity)) OR global temperature* OR (temperature* adj2 (increase* OR variab* OR trend*)) OR “extreme weather” OR “extreme temperature” OR (heatwave* OR heat-wave* OR "heat wave") OR (cold adj1 extreme*) OR (rise adj4 (sea level OR sea-level OR sealevel)) OR flood* OR “storm surge” OR landslide* OR (wildfire* OR wild-fire* OR wild fire) OR windstorm* OR cyclone* OR (humidit* adj2 (increas* OR change* OR elevation* OR variabilit* OR pattern*)) OR “rainfall pattern*” OR ((acid OR acidifi*) adj1 rain*) OR “carbon footprint” OR (ozone* adj2 depletion) OR “climate pollutant*” OR “extended drought*” OR “cloud cover” OR (change adj2 ("land use" OR "land cover")) OR “soil erosion” OR “soil quality” OR (nutrient* adj1 (run-off OR runoff OR "run off")) OR ((quality OR availability) adj1 (groundwater OR ground-water OR "ground water" OR freshwater OR fresh-water OR "fresh water" OR surfacewater OR surface-water OR "surface water" OR "irrigation water")) OR aquifer* OR (water adj2 (availab* OR security OR stress OR scarcity)) OR (water adj2 quality) OR biodivers* OR “biological divers*” OR pollinator* OR ((bee OR bees OR insect OR insects) adj2 decline) OR “ecosystem* change*” OR "health of the planet" OR “environment* change*” OR (chemical adj1 contamination) OR (pest* adj4 (crop* OR plant*)) OR (plant* adj1 (pathogen* OR disease*))).ti,ab.

2. (pulses OR banana* OR tomato* OR Orange* OR mandarin* OR apple* OR grapes OR plantain* OR Coconut* OR Pineapple* OR Lemon* OR Lime* OR citrus OR Grapefruit* OR Dates OR Olive* OR berries OR strawberr* OR raspberr* blackberr* OR melon* OR Cherry OR cherries OR Kiwi* OR plum* OR Pomegranate OR Peach* OR avocado* OR papaya* OR pumpkin* OR onion* OR bean* OR Peas OR Pepper* OR piment* OR Spinach OR Lettuce OR kale OR garlic OR broccoli OR sprout*).ti,ab.

3. ((quality adj2 (crop OR vegetable OR fruit OR yield)) OR (taste adj2 (crop OR vegetable OR fruit)) OR ((micronutrient* OR macronutrient* OR nutrient* OR nutrition* OR nutritious OR nutriti* OR vitamin* OR mineral OR iron OR zinc OR calcium) adj2 (value* OR content* OR composition*

OR concentration* OR quality)) OR beta-carotene OR "beta carotene" OR betacarotene OR folate* OR "folic acid*" OR "folic-acid*" OR folicacid* OR "amino acid*" OR "amino-acid*" OR aminoacid* OR Carotenoids).ti,ab.

CAB Abstracts

1. ((climate adj1 (change* OR variability OR disruption)) OR (land* adj1 degradation) OR ((increas* OR elevat* OR fertili?ation) adj2 (carbon-dioxide OR carbondioxide OR "carbon dioxide" OR co2)) OR (soil adj2 (fertili?ation OR eutrophication)) OR “global warming” OR “greenhouse effect*” OR ((water OR soil*) adj4 (sodium OR saline?ation OR saline OR salinity)) OR global temperature* OR (temperature* adj2 (increase* OR variab* OR trend*)) OR “extreme weather” OR “extreme temperature” OR (heatwave* OR heat-wave* OR "heat wave") OR (cold adj1 extreme*) OR (rise adj4 (sea level OR sea-level OR sealevel)) OR flood* OR “storm surge” OR landslide* OR (wildfire* OR wild-fire* OR wild fire) OR windstorm* OR cyclone* OR (humidit* adj2 (increas* OR change* OR elevation* OR variabilit* OR pattern*)) OR “rainfall pattern*” OR ((acid OR acidifi*) adj1 rain*) OR “carbon footprint” OR (ozone* adj2 depletion) OR “climate pollutant*” OR “extended drought*” OR “cloud cover” OR (change adj2 ("land use" OR "land cover")) OR “soil erosion” OR “soil quality” OR (nutrient* adj1 (run-off OR runoff OR "run off")) OR ((quality OR availability) adj1 (groundwater OR ground-water OR "ground water" OR freshwater OR fresh-water OR "fresh water" OR surfacewater OR surface-water OR "surface water" OR "irrigation water")) OR aquifer* OR (water adj2 (availab* OR security OR stress OR scarcity)) OR (water adj2 quality) OR biodivers* OR “biological divers*” OR pollinator* OR ((bee OR bees OR insect OR insects) adj2 decline) OR “ecosystem* change*” OR "health of the planet" OR “environment* change*” OR (chemical adj1 contamination) OR (pest* adj4 (crop* OR plant*)) OR (plant* adj1 (pathogen* OR disease*))).ti,ab.

2. (pulses OR banana* OR tomato* OR Orange* OR mandarin* OR apple* OR grapes OR plantain* OR Coconut* OR Pineapple* OR Lemon* OR Lime* OR citrus OR Grapefruit* OR Dates OR Olive* OR berries OR strawberr* OR raspberr* blackberr* OR melon* OR Cherry OR cherries OR Kiwi* OR plum* OR Pomegranate OR Peach* OR avocado* OR papaya* OR pumpkin* OR onion* OR bean* OR Peas OR Pepper* OR piment* OR Spinach OR Lettuce OR kale OR garlic OR broccoli OR sprout*).ti,ab.

3. ((quality adj2 (crop OR vegetable OR fruit OR yield)) OR (taste adj2 (crop OR vegetable OR fruit)) OR ((micronutrient* OR macronutrient* OR nutrient* OR nutrition* OR nutritious OR nutriti* OR vitamin* OR mineral OR iron OR zinc OR calcium) adj2 (value* OR content* OR composition* OR concentration* OR quality)) OR beta-carotene OR "beta carotene" OR betacarotene OR folate* OR "folic acid*" OR "folic-acid*" OR folicacid* OR "amino acid*" OR "amino-acid*" OR aminoacid* OR Carotenoids)).ti,ab.

GreenFILE

1. (climate N1 (change* OR variability OR disruption)) OR (land* N1 degradation) OR ((increas* OR elevat* OR fertilisation OR fertilization) N2 (carbon-dioxide OR carbondioxide OR "carbon dioxide" OR co2)) OR (soil N2 (fertilisation OR fertilization OR eutrophication)) OR “global warming” OR “greenhouse effect*” OR ((water OR soil*) N4 (sodium OR salinisation OR salinization OR saline OR salinity)) OR global temperature* OR (temperature* N2 (increase* OR variab* OR trend*)) OR “extreme weather” OR “extreme temperature” OR (heatwave* OR heat-wave* OR "heat wave") OR (cold N1 extreme*) OR (rise N4 (sea level OR sea-level OR sealevel)) OR flood* OR “storm surge” OR landslide* OR (wildfire* OR wild-fire* OR wild fire) OR windstorm* OR cyclone* OR (humidit* N2 (increas* OR change* OR elevation* OR variabilit* OR pattern*)) OR “rainfall pattern*” OR ((acid OR acidifi*) N1 rain*) OR “carbon footprint” OR (ozone* N2 depletion) OR “climate pollutant*” OR “extended drought*” OR “cloud cover” OR (change N2 ("land use" OR "land cover")) OR “soil erosion” OR “soil quality” OR (nutrient* N1 (run-off OR runoff OR "run off")) OR ((quality OR availability) N1 (groundwater OR ground-water

OR "ground water" OR freshwater OR fresh-water OR "fresh water" OR surfacewater OR surface-water OR "surface water" OR "irrigation water")) OR aquifer* OR (water N2 (availab* OR security OR stress OR scarcity)) OR (water N2 quality) OR biodivers* OR “biological divers*” OR pollinator* OR ((bee OR bees OR insect OR insects) N2 decline) OR “ecosystem* change*” OR "health of the planet" OR “environment* change*” OR (chemical N1 contamination) OR (pest* N4 (crop* OR plant*)) OR (plant* N1 (pathogen* OR disease*))

2. pulses OR banana* OR tomato* OR Orange* OR mandarin* OR apple* OR grapes OR plantain* OR Coconut* OR Pineapple* OR Lemon* OR Lime* OR citrus OR Grapefruit* OR Dates OR Olive* OR berries OR strawberr* OR raspberr* blackberr* OR melon* OR Cherry OR cherries OR Kiwi* OR plum* OR Pomegranate OR Peach* OR avocado* OR papaya* OR pumpkin* OR onion* OR bean* OR Peas OR Pepper* OR piment* OR Spinach OR Lettuce OR kale OR garlic OR broccoli OR sprout*

3. (quality N2 (crop OR vegetable OR fruit OR yield)) OR (taste N2 (crop OR vegetable OR fruit)) OR ((micronutrient* OR macronutrient* OR nutrient* OR nutrition* OR nutritious OR nutriti* OR vitamin* OR mineral OR iron OR zinc OR calcium) N2 (value* OR content* OR composition* OR concentration* OR quality)) OR beta-carotene OR "beta carotene" OR betacarotene OR folate* OR "folic acid*" OR "folic-acid*" OR folicacid* OR "amino acid*" OR "amino-acid*" OR aminoacid* OR Carotenoids

Web of Science

1. TS=(((climate near/1 (change* OR variability OR disruption)) OR (land* near/1 degradation) OR ((increas* OR elevat* OR fertili?ation) near/2 (carbon-dioxide OR carbondioxide OR "carbon dioxide" OR co2)) OR (soil near/2 (fertili?ation OR eutrophication)) OR “global warming” OR “greenhouse effect*” OR ((water OR soil*) near/4 (sodium OR salini?ation OR saline OR salinity)) OR global temperature* OR (temperature* near/2 (increase* OR variab* OR trend*)) OR “extreme weather” OR “extreme temperature” OR (heatwave* OR heat-wave* OR "heat wave") OR (cold near/1 extreme*) OR (rise near/4 (sea level OR sea-level OR sealevel)) OR flood* OR “storm surge” OR landslide* OR (wildfire* OR wild-fire* OR wild fire) OR windstorm* OR cyclone* OR (humidit* near/2 (increas* OR change* OR elevation* OR variabilit* OR pattern*)) OR “rainfall pattern*” OR ((acid OR acidifi*) near/1 rain*) OR “carbon footprint” OR (ozone* near/2 depletion) OR “climate pollutant*” OR “extended drought*” OR “cloud cover” OR (change near/2 ("land use" OR "land cover")) OR “soil erosion” OR “soil quality” OR (nutrient* near/1 (run-off OR runoff OR "run off")) OR ((quality OR availability) near/1 (groundwater OR ground-water OR "ground water" OR freshwater OR fresh-water OR "fresh water" OR surfacewater OR surface-water OR "surface water" OR "irrigation water")) OR aquifer* OR (water near/2 (availab* OR security OR stress OR scarcity)) OR (water near/2 quality) OR biodivers* OR “biological divers*” OR pollinator* OR ((bee OR bees OR insect OR insects) near/2 decline) OR “ecosystem* change*” OR "health of the planet" OR “environment* change*” OR (chemical near/1 contamination) OR (pest* near/4 (crop* OR plant*)) OR (plant* near/1 (pathogen* OR disease*)))

2. TS=(pulses OR banana* OR tomato* OR Orange* OR mandarin* OR apple* OR grapes OR plantain* OR Coconut* OR Pineapple* OR Lemon* OR Lime* OR citrus OR Grapefruit* OR Dates OR Olive* OR berries OR strawberr* OR raspberr* blackberr* OR melon* OR Cherry OR cherries OR Kiwi* OR plum* OR Pomegranate OR Peach* OR avocado* OR papaya* OR pumpkin* OR onion* OR bean* OR Peas OR Pepper* OR piment* OR Spinach OR Lettuce OR kale OR garlic OR broccoli OR sprout*)

3. TS=((quality near/2 (crop OR vegetable OR fruit OR yield)) OR (taste near/2 (crop OR vegetable OR fruit)) OR ((micronutrient* OR macronutrient* OR nutrient* OR nutrition* OR nutritious OR nutriti* OR vitamin* OR mineral OR iron OR zinc OR calcium) near/2 (value* OR content* OR composition* OR concentration* OR quality)) OR beta-carotene OR "beta carotene" OR betacarotene

OR folate* OR "folic acid*" OR "folic-acid*" OR folicacid OR "amino acid*" OR "amino-acid*" OR aminoacid* OR Carotenoids)

Scopus

1. TITLE-ABS-KEY ((climate W/2 (change* OR variability OR disruption)) OR (land* W/1 degradation) OR ((increas* OR elevat* OR fertili?ation) W/2 (carbon-dioxide OR carbondioxide OR "carbon dioxide" OR co2)) OR (soil W/2 (fertili?ation OR eutrophication)) OR "global warming" OR "greenhouse effect*" OR ((water OR soil*) W/4 (sodium OR salini?ation OR saline OR salinity)) OR "global temperature*" OR (temperature* W/2 (increas* OR variab* OR trend*)) OR "extreme weather" OR "extreme temperature*" OR (heatwave* OR heat-wave* OR "heat wave") OR (cold W/1 extreme*) OR ((rise OR increase) W/4 ("sea level" OR "sea-level" OR sealevel)) OR flood* OR "storm surge*" OR landslide* OR (wildfire* OR wild-fire* OR "wild fire*") OR windstorm* OR cyclone* OR (humidit* W/2 (increas* OR change* OR elevation* OR variabilit* OR pattern*)) OR "rainfall pattern*" OR ((acid OR acidifi*) W/1 rain*) OR "carbon footprint" OR (ozone* W/2 depletion) OR "climate pollutant*" OR "extended drought*" OR "cloud cover" OR (change W/2 ("land use" OR "land cover")) OR "soil erosion" OR "soil quality" OR (nutrient* W/1 ("run-off" OR runoff OR "run off")) OR ((quality OR availability) W/1 (groundwater OR ground-water OR "ground water" OR freshwater OR fresh-water OR "fresh water" OR surfacewater OR surface-water OR "surface water" OR "irrigation water")) OR aquifer* OR (water W/2 (availab* OR security OR stress OR scarcity)) OR (water W/2 quality) OR biodivers* OR "biological divers*" OR pollinator* OR ((bee OR bees OR insect OR insects) W/2 decline) OR (ecosystem* W/2 change*) OR “health of the planet” OR (environment* W/2 change*) OR (chemical* W/1 contamination) OR (pest* W/4 (crop* OR plant*)) OR (plant* W/1 (pathogen* OR disease*)))

2. (pulses OR banana* OR tomato* OR Orange* OR mandarin* OR apple* OR grapes OR plantain* OR Coconut* OR Pineapple* OR Lemon* OR Lime* OR citrus OR Grapefruit* OR Dates OR Olive* OR berries OR strawberr* OR raspberr* blackberr* OR melon* OR Cherry OR cherries OR Kiwi* OR plum* OR Pomegranate OR Peach* OR avocado* OR papaya* OR pumpkin* OR onion* OR bean* OR Peas OR Pepper* OR piment* OR Spinach OR Lettuce OR kale OR garlic OR broccoli OR sprout*)

3. TITLE-ABS-KEY ((quality W/2 (crop OR vegetable OR fruit OR yield)) OR (taste W/2 (crop OR vegetable OR fruit)) OR ((micronutrient* OR macronutrient* OR nutrient* OR nutrition* OR nutritious OR nutriti* OR vitamin* OR mineral OR iron OR zinc OR calcium) W/2 (value* OR content* OR composition* OR concentration* OR quality)) OR beta-carotene OR "beta carotene" OR betacarotene OR folate* OR "folic acid*" OR "folic-acid*" OR folicacid* OR "amino acid*" OR "amino-acid*" OR aminoacid* OR Carotenoids)

EMBASE

1. ((climate adj1 (change* OR variability OR disruption)) OR (land* adj1 degradation) OR ((increas* OR elevat* OR fertili#ation) adj2 (carbon-dioxide OR carbondioxide OR "carbon dioxide" OR co2)) OR (soil adj2 (fertili#ation OR eutrophication)) OR global warming OR greenhouse effect* OR ((water OR soil*) adj4 (sodium OR salini#ation OR saline OR salinity)) OR global temperature* OR (temperature* adj2 (increase* OR variab* OR trend*)) OR extreme weather OR extreme temperature OR (heatwave* OR heat-wave* OR "heat wave") OR (cold adj1 extreme*) OR (rise adj4 (sea level OR sea-level OR sealevel)) OR flood* OR storm surge* OR landslide* OR (wildfire* OR wild-fire* OR wild fire) OR windstorm* OR cyclone* OR (humidit* adj2 (increas* OR change* OR elevation* OR variabilit* OR pattern*)) OR rainfall pattern* OR ((acid OR acidifi*) adj1 rain*) OR carbon footprint OR (ozone* adj2 depletion) OR climate pollutant* OR extended drought* OR cloud cover OR (change adj2 ("land use" or "land cover")) OR soil erosion OR soil quality OR (nutrient* adj1 (run-off or runoff or "run off")) OR ((quality OR availability) adj1 (groundwater OR ground-water OR "ground water" OR freshwater OR fresh-water OR "fresh water" OR surfacewater OR surface-

water OR "surface water" OR "irrigation water")) OR aquifer* OR (water adj2 (availab* OR security OR stress OR scarcity)) OR (water adj2 quality) OR biodivers* OR biological divers* OR pollinator* OR ((bee OR bees OR insect OR insects) adj2 decline) OR ecosystem* change* OR "health of the planet" OR environment* change* OR (chemical adj1 contamination) OR (pest* adj4 (crop* OR plant*)) OR (plant* adj1 (pathogen* OR disease*))).ti,ab.

2. (pulses OR banana* OR tomato* OR Orange* OR mandarin* OR apple* OR grapes OR plantain* OR Coconut* OR Pineapple* OR Lemon* OR Lime* OR citrus OR Grapefruit* OR Dates OR Olive* OR berries OR strawberr* OR raspberr* blackberr* OR melon* OR Cherry OR cherries OR Kiwi* OR plum* OR Pomegranate OR Peach* OR avocado* OR papaya* OR pumpkin* OR onion* OR bean* OR Peas OR Pepper* OR piment* OR Spinach OR Lettuce OR kale OR garlic OR broccoli OR sprout*).ti,ab.

3. ((quality W/2 (crop OR vegetable OR fruit OR yield)) OR (taste W/2 (crop OR vegetable OR fruit)) OR ((micronutrient* OR macronutrient* OR nutrient* OR nutrition* OR nutritious OR nutriti* OR vitamin* OR mineral OR iron OR zinc OR calcium) W/2 (value* OR content* OR composition* OR concentration* OR quality)) OR beta-carotene OR "beta carotene" OR betacarotene OR folate* OR "folic acid*" OR "folic-acid*" OR folicacid* OR "amino acid*" OR "amino-acid*" OR aminoacid* OR Carotenoids).ti,ab.

MEDLINE

1. ((climate adj1 (change* OR variability OR disruption)) OR (land* adj1 degradation) OR ((increas* OR elevat* OR fertili#ation) adj2 (carbon-dioxide OR carbondioxide OR "carbon dioxide" OR co2)) OR (soil adj2 (fertili#ation OR eutrophication)) OR global warming OR greenhouse effect* OR ((water OR soil*) adj4 (sodium OR salini#ation OR saline OR salinity)) OR global temperature* OR (temperature* adj2 (increase* OR variab* OR trend*)) OR extreme weather OR extreme temperature OR (heatwave* OR heat-wave* OR "heat wave") OR (cold adj1 extreme*) OR (rise adj4 (sea level OR sea-level OR sealevel)) OR flood* OR storm surge* OR landslide* OR (wildfire* OR wild-fire* OR wild fire) OR windstorm* OR cyclone* OR (humidit* adj2 (increas* OR change* OR elevation* OR variabilit* OR pattern*)) OR rainfall pattern* OR ((acid OR acidifi*) adj1 rain*) OR carbon footprint OR (ozone* adj2 depletion) OR climate pollutant* OR extended drought* OR cloud cover OR (change adj2 ("land use" or "land cover")) OR soil erosion OR soil quality OR (nutrient* adj1 (run-off or runoff or "run off")) OR ((quality OR availability) adj1 (groundwater OR ground-water OR "ground water" OR freshwater OR fresh-water OR "fresh water" OR surfacewater OR surface-water OR "surface water" OR "irrigation water")) OR aquifer* OR (water adj2 (availab* OR security OR stress OR scarcity)) OR (water adj2 quality) OR biodivers* OR biological divers* OR pollinator* OR ((bee OR bees OR insect OR insects) adj2 decline) OR ecosystem* change* OR "health of the planet" OR environment* change* OR (chemical adj1 contamination) OR (pest* adj4 (crop* OR plant*)) OR (plant* adj1 (pathogen* OR disease*))).ti,ab.

2. (pulses OR banana* OR tomato* OR Orange* OR mandarin* OR apple* OR grapes OR plantain* OR Coconut* OR Pineapple* OR Lemon* OR Lime* OR citrus OR Grapefruit* OR Dates OR Olive* OR berries OR strawberr* OR raspberr* blackberr* OR melon* OR Cherry OR cherries OR Kiwi* OR plum* OR Pomegranate OR Peach* OR avocado* OR papaya* OR pumpkin* OR onion* OR bean* OR Peas OR Pepper* OR piment* OR Spinach OR Lettuce OR kale OR garlic OR broccoli OR sprout*).ti,ab.

3. ((quality W/2 (crop OR vegetable OR fruit OR yield)) OR (taste W/2 (crop OR vegetable OR fruit)) OR ((micronutrient* OR macronutrient* OR nutrient* OR nutrition* OR nutritious OR nutriti* OR vitamin* OR mineral OR iron OR zinc OR calcium) W/2 (value* OR content* OR composition* OR concentration* OR quality)) OR beta-carotene OR "beta carotene" OR betacarotene OR folate* OR "folic acid*" OR "folic-acid*" OR folicacid* OR "amino acid*" OR "amino-acid*" OR aminoacid* OR Carotenoids).ti,ab.

iii. Nuts & Seeds Yield

Web of Science

1. TS=(nut* OR seed* OR cashew* OR "cashew nut" OR peanut* OR walnut* OR pistachio* OR "pistachio nut*" OR hazelnut* OR "sunflower seed*" OR chestnut* OR pecan* OR "pecan nut*" OR groundnut* OR macadamia* OR "macadamia nut*" OR almond* OR "pine nut*" OR hempseed* OR "hemp seed*" OR sheanut* OR "kola nut*" OR linseed* OR rapeseed* OR "chia seed*")

2. TS=((pest* near/4 (crop* OR plant* OR tree*)) OR (chemical* near/1 contamination) OR (environment* near/2 (change* OR damage*)) OR (ecosystem* near/2 change*) OR ((bee OR bees OR insect OR insects) near/2 decline) OR (pollinator* near/2 (loss OR deplet* OR declin*)) OR pollinator* OR (environment* near/2 chang*) OR "environmental stressor*" OR "environmental change*" OR (biodiversity near/2 (loss OR deplet* OR decreas*)) OR (water near/2 (availab* OR quality OR stress OR security OR scarcity OR deplet*)) OR ((quality OR availability) near/1 (groundwater OR ground-water OR "ground water" OR freshwater OR fresh-water OR "fresh water" OR "surface water" OR surface-water OR "irrigation water")) OR ((rise* OR increas* OR elevated OR elevat*) near/2 ("UV" OR "ultraviolet" OR ultra-violet OR "UV radiation" OR "ultraviolet radiation" OR "ultra-violet radiation")) OR ("atmospheric CO2" OR "atmospheric carbon dioxide" OR "atmospheric carbon-dioxide") OR ((rise* OR elevated OR elevation OR increas*) near/2 "tropospheric ozone") OR "tropospheric ozone" OR "soil erosion" OR "soil quality" OR (change near/2 ("land use" OR "land cover")) OR ((acid OR acidifi*) near/1 rain*) OR (ozone near/2 depletion) OR (humidit* near/2 (increas* OR change* OR elevation OR variabilit* OR pattern*)) OR "extended drought*" OR "extreme drought*" OR "climate pollutant" OR "carbon footprint" OR "rainfall pattern*" OR windstorm* OR "storm surge" OR "landslide" OR ((rise* OR increase*) near/4 ("sea level" OR sea-level OR sealevel)) OR cyclone* OR (cold near/1 extreme*) OR flood* OR (heatwave* OR heat-wave* OR "heat wave*") OR (temperature near/2 (elevated OR elevation OR rise OR rising OR increas*)) OR "extreme temperature" OR "extreme weather" OR ((water or soil*) near/4 (sodium or salini?ation or saline or salinity)) OR "greenhouse effect" OR (soil near/2 (fertili?ation OR eutrophication)) OR "global warming" OR ((increas* OR elevat* OR fertili?ation) near/2 (carbon-dioxide OR carbondioxide OR "carbon dioxide" OR CO2)) OR (land* near/1 degradation) OR (climate near/2 (change* OR variability OR disruption)))

3. TS=("change in yield" OR "yield change" OR "crop production" OR "crop growth" OR productivity OR production OR growth OR yield OR harvest OR biomass OR "dry weight" OR dry-weight OR "fresh weight" OR fresh-weight)

MEDLINE

1. (nut* OR seed* OR cashew* OR “cashew nut*” OR peanut* OR walnut* OR pistachio* OR "pistachio nut*" OR hazelnut* OR "sunflower seed*" OR chestnut* OR pecan* OR "pecan nut*" OR groundnut* OR macadamia* OR "macadamia nut*" OR almond* OR "pine nut*" OR hempseed* OR "hemp seed*" OR sheanut* OR "kola nut*" OR linseed* OR rapeseed* OR "chia seed*").ti,ab.

2. ((pest* adj4 (crop* OR plant* OR tree*)) OR (chemical* adj1 contamination) OR (environment* adj2 (change* or damage*)) OR (ecosystem* adj2 change*) OR ((bee OR bees OR insect OR insects) adj2 decline) OR (pollinator* adj2 (loss OR deplet* OR declin*)) OR pollinator* OR (environment* adj2 chang*) OR "environmental stressor*" OR "environmental change*" OR (biodiversity adj2 (loss OR deplet* OR decreas*)) OR (water adj2 (availab* OR quality OR stress OR security OR scarcity OR deplet*)) OR ((quality OR availability) adj1 (groundwater OR ground-water OR "ground water" OR freshwater OR fresh-water OR "fresh water" OR "surface water" OR surface-water OR "irrigation water")) OR ((rise* OR increas* OR elevated OR elevat*) adj2 ("UV" OR "ultraviolet" OR ultra-violet OR "UV radiation" OR "ultraviolet radiation" OR "ultra-violet radiation")) OR ("atmospheric CO2" OR "atmospheric carbon dioxide" OR "atmospheric carbon-dioxide") OR ((rise* OR elevated OR elevation OR increas*) adj2 "tropospheric ozone") OR "tropospheric ozone" OR "soil erosion"

OR "soil quality" OR (change adj2 ("land use" OR "land cover")) OR ((acid OR acidifi*) adj1 rain*) OR (ozone adj2 depletion) OR (humidit* adj2 (increas* OR change* OR elevation OR variabilit* OR pattern*)) OR "extended drought*" OR "extreme drought*" OR "climate pollutant*" OR "carbon footprint" OR "rainfall pattern*" OR windstorm* OR "storm surge" OR landslide OR ((rise* OR increase*) adj4 ("sea level" OR sea-level OR sealevel)) OR cyclone* OR (cold adj1 extreme*) OR flood* OR (heatwave* OR heat-wave* OR "heat wave*") OR (temperature adj2 (elevated OR elevation OR rise OR rising OR increas*)) OR "extreme temperature*" OR "extreme weather" OR ((water or soil*) adj4 (sodium or salini#ation or saline or salinity)) OR "greenhouse effect" (soil adj2 (fertili#ation OR eutrophication)) OR "global warming" OR ((increas* OR elevat* OR fertili#ation) adj2 (carbon-dioxide OR carbondioxide OR "carbon dioxide" OR CO2)) OR (land* adj1 degradation) OR (climate adj2 (change* OR variability OR disruption))).ti,ab.

3. ((yield adj3 change) OR "crop production" OR "crop growth" OR producti* OR growth OR yield OR harvest OR biomass OR dry weight).ti,ab.

EMBASE

1. (nut* OR seed* OR cashew* OR “cashew nut*” OR peanut* OR walnut* OR pistachio* OR "pistachio nut*" OR hazelnut* OR "sunflower seed*" OR chestnut* OR pecan* OR "pecan nut*" OR groundnut* OR macadamia* OR "macadamia nut*" OR almond* OR "pine nut*" OR hempseed* OR "hemp seed*" OR sheanut* OR "kola nut*" OR linseed* OR rapeseed* OR "chia seed*").ti,ab.

2. ((pest* adj4 (crop* OR plant* OR tree*)) OR (chemical* adj1 contamination) OR (environment* adj2 (change* or damage*)) OR (ecosystem* adj2 change*) OR ((bee OR bees OR insect OR insects) adj2 decline) OR (pollinator* adj2 (loss OR deplet* OR declin*)) OR pollinator* OR (environment* adj2 chang*) OR "environmental stressor*" OR "environmental change*" OR (biodiversity adj2 (loss OR deplet* OR decreas*)) OR (water adj2 (availab* OR quality OR stress OR security OR scarcity OR deplet*)) OR ((quality OR availability) adj1 (groundwater OR ground-water OR "ground water" OR freshwater OR fresh-water OR "fresh water" OR "surface water" OR surface-water OR "irrigation water")) OR ((rise* OR increas* OR elevated OR elevat*) adj2 ("UV" OR "ultraviolet" OR ultra-violet OR "UV radiation" OR "ultraviolet radiation" OR "ultra-violet radiation")) OR ("atmospheric CO2" OR "atmospheric carbon dioxide" OR "atmospheric carbon-dioxide") OR ((rise* OR elevated OR elevation OR increas*) adj2 "tropospheric ozone") OR "tropospheric ozone" OR "soil erosion" OR "soil quality" OR (change adj2 ("land use" OR "land cover")) OR ((acid OR acidifi*) adj1 rain*) OR (ozone adj2 depletion) OR (humidit* adj2 (increas* OR change* OR elevation OR variabilit* OR pattern*)) OR "extended drought*" OR "extreme drought*" OR "climate pollutant*" OR "carbon footprint" OR "rainfall pattern*" OR windstorm* OR "storm surge" OR landslide OR ((rise* OR increase*) adj4 ("sea level" OR sea-level OR sealevel)) OR cyclone* OR (cold adj1 extreme*) OR flood* OR (heatwave* OR heat-wave* OR "heat wave*") OR (temperature adj2 (elevated OR elevation OR rise OR rising OR increas*)) OR "extreme temperature*" OR "extreme weather" OR ((water or soil*) adj4 (sodium or salini#ation or saline or salinity)) OR "greenhouse effect" (soil adj2 (fertili#ation OR eutrophication)) OR "global warming" OR ((increas* OR elevat* OR fertili#ation) adj2 (carbon-dioxide OR carbondioxide OR "carbon dioxide" OR CO2)) OR (land* adj1 degradation) OR (climate adj2 (change* OR variability OR disruption))).ti,ab.

3. ((yield adj3 change) OR "crop production" OR "crop growth" OR producti* OR growth OR yield OR harvest OR biomass OR dry weight).ti,ab.

GreenFILE

1. (nut* OR seed* OR cashew* OR "cashew nut" OR peanut* OR walnut* OR pistachio* OR "pistachio nut*" OR hazelnut* OR "sunflower seed*" OR chestnut* OR pecan* OR "pecan nut*" OR groundnut* OR macadamia* OR "macadamia nut*" OR almond* OR "pine nut*" OR hempseed* OR "hemp seed*" OR sheanut* OR "kola nut*" OR linseed* OR rapeseed* OR "chia seed*")

2. ((pest* N4 (crop* OR plant* OR tree*)) OR (chemical* N1 contamination) OR (environment* N2 (change* OR damage*)) OR (ecosystem* N2 change*) OR ((bee OR bees OR insect OR insects) N2 decline) OR (pollinator* N2 (loss OR deplet* OR declin*)) OR pollinator* OR (environment* N2 chang*) OR "environmental stressor*" OR "environmental change*" OR (biodiversity N2 (loss OR deplet* OR decreas*)) OR (water N2 (availab* OR quality OR stress OR security OR scarcity OR deplet*)) OR ((quality OR availability) N1 (groundwater OR ground-water OR "ground water" OR freshwater OR fresh-water OR "fresh water" OR "surface water" OR surface-water OR "irrigation water")) OR ((rise* OR increas* OR elevated OR elevat*) N2 ("UV" OR "ultraviolet" OR ultra-violet OR "UV radiation" OR "ultraviolet radiation" OR "ultra-violet radiation")) OR ("atmospheric CO2" OR "atmospheric carbon dioxide" OR "atmospheric carbon-dioxide") OR ((rise* OR elevated OR elevation OR increas*) N2 "tropospheric ozone") OR "tropospheric ozone" OR "soil erosion" OR "soil quality" OR (change N2 ("land use" OR "land cover")) OR ((acid OR acidifi*) N1 rain*) OR (ozone N2 depletion) OR (humidit* N2 (increas* OR change* OR elevation OR variabilit* OR pattern*)) OR "extended drought*" OR "extreme drought*" OR "climate pollutant" OR "carbon footprint" OR "rainfall pattern*" OR windstorm* OR "storm surge" OR "landslide" OR ((rise* OR increase*) N4 ("sea level" OR sea-level OR sealevel)) OR cyclone* OR (cold N1 extreme*) OR flood* OR (heatwave* OR heat-wave* OR "heat wave*") OR (temperature N2 (elevated OR elevation OR rise OR rising OR increas*)) OR "extreme temperature" OR "extreme weather" OR ((water or soil*) N4 (sodium or salini?ation or saline or salinity)) OR "greenhouse effect" OR (soil N2 (fertili?ation OR eutrophication)) OR "global warming" OR ((increas* OR elevat* OR fertili?ation) N2 (carbon-dioxide OR carbondioxide OR "carbon dioxide" OR CO2)) OR (land* N1 degradation) OR (climate N2 (change* OR variability OR disruption)))

3. ((yield N3 change) OR "crop production" OR "crop growth" OR producti* OR growth OR yield OR harvest OR biomass OR dry weight).

B. Modified CASP checklist

Modified checklist derived from the Critical Appraisal Skills Programme for randomised controlled trials

#	Criterion Description	Issues Considered
1	Clear study description	• Did the authors provide a clear description of the crops evaluated?<br>• Did the authors provide a clear description of (combination of) environmental changes, including levels/concentrations evaluated?<br>• Was a clear description given of crops, field / greenhouse conditions?<br>• Did they give a clear justification of study in particular area – including a description of environmental conditions and trends?
2	Appropriate comparison group/situation	• Were crops under the "intervention" compared to an appropriate and comparable baseline group/situation?
3	Clear methods description	• Were the methods of measuring exposure clearly described?<br>• Were the methods of measuring outcome clearly described?
4	Rigorous and clearly described analysis	• Are sufficient data presented to support the findings?<br>• Were analyses described in detail? (Could they be repeated by someone not involved in the study)<br>• Did the researchers critically examine their potential biases during measurement, analysis and selection of data for presentation?

For inclusion in meta-analysis:

5	Precision of measure of effect	• What are the confidence limits?<br>• Were they statistically significant?

C. Overview of the included studies in the systematic review

	Author & Year	Title	Study design	Location	Crop Type	Crop Group	Environmental Exposure	No. of Experiments	Change in Exposure (min, max)	Yield/Nutritional quality	% Change in Outcome (min, max)	Total Quality Score
1	Abdel-Aziz and Sadik 2017	Effect of water and salt stresses on productivity of cantaloupe in Ismailia soil	Field	Egypt	Cantaloupe Melon	Cucurbits	Water availability	3	-30, -10 % ETc	Yield	-46.0, -3.4	4
							Water salinity	3	+0.8, +5.5 dS/m	Yield	-44.4, -1.4	
							Water availability & salinity	9	-30, -10 % ETc & +0.8, +5.5 dS/m	Yield	-77.6, -6.7	
2	Abu-Awwad AM 2001	Influence of different water quantities and qualities on lemon trees and soil salt distribution at the Jordan Valley	Field	Jordan	Lemon	Citrus	Water availability	8	-100, -25 %ETc	Yield	-100, -1.8	4
							Water salinity	4	+1.8, +3.6 dS/m	Yield	-73.5, -18.2	
3	Akrami and Arzani 2018	"Physiological alterations due to field salinity stress in melon (Cucumis melo L.)"	Field	Iran	Melon	Cucurbits	Water salinity	1	+7.3 dS/m	Yield	-42.7	4
4	Atay et al 2017	Investigating effects of over-irrigation and deficit irrigation on yield and fruit quality in pink lady^{tm} 'Rosy Glow' apple	Field	France	Apple	Pome	Water availability	1	-25 %	Yield	-13.1	4
5	Aydinsakir et al 2016	Assessment of different irrigation levels on peanut crop yield and quality components under Mediterranean conditions	Field	Turkey	Peanut	Nut	Water availability	8	-630, -154 mm	Yield	-83.0, -9.5	4
6	Bannayan et al 2009	Interactive effects of elevated CO_2 and temperature on growth and development of a short- and long-season peanut cultivar	Growth chamber	USA	Peanut	Nut	CO_2	2	+300 µmol/mol	Yield	-53.3, -24.4	4
						Nut	Temperature	4	+2.5, 5 °C		-94.6, -36.3	
						Nut	CO_2 & temperature	4	+300 µmol/mol & +2.5, 5 °C		-91.6, -45.6	

7	Besset et al 2001	Effect of water stress applied during the final stage of rapid growth on peach trees (cv. Big-Top)	Field	France	Peach	Drupe	Water availability	4	-90, -17 L/day	Yield	-29.1, +21.2	4
8	Bezerra et al 2018	Physiological alterations and production of guava under water salinity and nitrogen fertilizer application	Field	Brazil	Guava	Berry	Salinity	4	+0.8, +3.2 dS/m	Yield	-16, -4	4
9	Bignami et al 1997	Influence of irrigation on the growth and production of young hazelnuts	Field	Italy	Hazelnut	Nut	Water availability	6	-104.6, -21.3 mm	Yield	-87.3, +14.1	4
10	Bignami et al 2009	Effects of irrigation on growth and yield components of hazelnut (Corylus avellana L.) in Central Italy.	Field	Italy	Hazelnut	Nut	Water availability	3	-135.2, -33.8 mm	Yield	-43.4, +23.4	4
11	Bindi et al 1996	The effect of elevated CO2 concentration on grapevine growth under field conditions	Field	Italy	Grape	Berry	CO_2	1	+341 ppm	Yield	+21.2	4
12	Bindi et al 2001	Free Air CO2 Enrichment (FACE) of grapevine (Vitis vinifera L.): II. Growth and quality of grape and wine in response to elevated CO2 concentrations	Field	Italy	Grape	Berry	CO_2	8	+200, +350 ppm	Yield	+17.2, 82.7	5
							CO_2	6	+200, +350 ppm	Flavonoids	+22.8, +41.7	
13	Bindi et al 2005	Physiological and yield responses of grapevine (Vitis vinifera L.) exposed to elevated CO2 concentrations in a Free Air CO2 enrichment (FACE)	Field	Italy	Grape	Berry	CO_2	2	+341, +339	Yield	+37.3, +44.3	4
14	Blaikie et al 2001	Productivity and water relations of field-grown cashew: a comparison of sprinkler and drip irrigation	Field	Australia	Cashew	Nut	Water availability	3	-500, -115 L/tree/week	Yield	-58.1, -21.6	4
15	Boem et al 1994	Some effects of soil salinity on growth, development and yield of rapeseed (Brassica napus L.)	Field	Argentina	Rapeseed	Seed	Water salinity	4	+2.2, +7.7 dS/m	Yield	-15.0, +21.0	5
16	Boland et al 1993	Effect of saline water combined with restricted irrigation on peach tree growth and water use	Field	Australia	Peach	Drupe	Water salinity	6	+0.15, +0.9 dS/m	Yield	-31.2, -2.9	4

17	Boman BJ 2005	Salinity effects on Florida grapefruit in the Indian river region	Field	USA	Grapefruit	Citrus	Water salinity	24	+1.6, +4.8 dS/m	Yield	-41.8, +8.6	4
18	Bozkurt et al 2015	Effects of deficit irrigation treatments on yield and plant growth of young apricot trees	Field	Turkey	Apricot	Drupe	Water availability	8	-100, -25 %ETc	Yield	-93.7, +29.8	4
19	Brito et al 2017	Physiological characteristics and yield of 'Perola' pineapple in the semi-arid region	Field	Brazil	Pineapple	Bromeliad	Water availability & salinity	2	-50, -75 %ETc & +2.85 dS/m	Yield	-64.3, -58.9	4
20	Buchner et al 2008	Effects of regulated deficit irrigation on walnut (Juglans regia) grafted on Northern California Black (Juglans hindsii) or Paradox rootstock	Field	USA	Walnut	Nut	Water availability	16	-5.8, -2.3 ml/ha	Yield	-43.5, -5.2	4
21	Buendia et al 2008	Effect of regulated deficit irrigation and crop load on the antioxidant compounds of peaches	Field	Spain	Peach	Drupe	Water availability	4	-75 %ETc	Vitamin C	-14.4, +15.5	4
							Water availability	8	-75 %ETc	Phenols	-32.9, +140.0	
							Water availability	4	-75 %ETc	Carotenoids	-33.3, -4.6	
							Water availability	10	-75 %ETc	Flavonoids	-63.3, +194.1	
22	Burkey et al 2007	Elevated carbon dioxide and ozone effects on peanut: II. Seed yield and quality	Field	USA	Peanut	Nut	CO_2	3	+173, +634 µmol/mol	Yield	+18.5, +25.8	5
							O_3	1	+29 nmol/mol		-27.0	
							CO_2 & O_3	2	+173, +355 µmol/mol & +29 nmol/mol		-0.9, +12.6	
23	Cabral et al 2018	Influence of irrigation on yield and quality of cv. Touriga Franca in the Douro region	Field	Portugal	Grape	Berry	Water availability	3	-304.7, -101.6 L	Yield	-29.2, -5.9	4
							Water availability	3	-304.7, -101.6 L	Phenols	-28.4, -17.9	

							Water availability	3	-304.7, -101.6 L	Flavonoids	-18.6, -7.3	
24	Calatayud et al 2006	Interactions between nitrogen fertilization and ozone in watermelon cultivar Reina de Corazones in open-top chambers. Effects on chlorophyll a fluorescence, lipid peroxidation and yield	Field	Spain	Watermelon	Cucurbits	O_3	2	+54, +77 nl/l	Yield	16.5, 156.0	4
25	Centofanti et al 2017	Deficit irrigation strategies and their impact on yield and nutritional quality of pomegranate fruit	Field	USA	Pomegranate	Pome	Water availability	6	-65, -25 %ETc	Yield	-25.4, +7.6	5
							Water availability	3	-65, -25 %ETc	Phenols	-20.5, -12.6	
							Water availability	27	-65, -25 %ETc	Flavonoids	-33.0, +12.2	
26	Chandler & Ferree 1990	Response of 'Raritan' and 'Surecrop' strawberry plants to drought stress	Greenhouse	USA	Strawberry	Berry	Water availability	2	-190 ml	Yield	-25.0, -6.0	4
27	Chartzoulakis et al 1999	Water use, growth, yield and fruit quality of 'Bonanza' oranges under different soil water regimes	Field	Greece	Orange	Citrus	Water availability	1	-36 %	Yield	+2.0	4
28	Chenafi et al 2016	Influence of irrigation strategies on productivity, fruit quality and soil-plant water status of subsurface drip-irrigated apple trees	Field	Switzerland	Apple	Pome	Water availability	6	-149, -66 Lm^{-2}	Yield	-18.9, +12.4	4
							Water availability	3	-149, -67 Lm^{-2}	Vitamin C	0.0, +14.3	
							Water availability	3	-149, -67 Lm^{-2}	Phenols	-1.1, +7.6	
							Water availability	3	-149, -67 Lm^{-2}	Flavonoids	0.0, +10.6	

29	Clifford et al 1993	The effects of elevated atmospheric carbon dioxide and water stress on light interception, dry matter production and yield in stands of groundnut (Arachis hypogaea L.)	Glasshouse	UK	Peanut	Nut	CO_2	2	+350 ppmv	Yield	+25.4, +25.6	5
30	Clifford et al 2000	Effects of elevated CO2, drought and temperature on the water relations and gas exchange of groundnut (Arachis hypogaea) stands grown in controlled environment glasshouses	Glasshouse	UK	Peanut	Nut	CO_2	1	+325 µmol /mol	Yield	+5.1	4
							Temperature	1	+4 ºC		-56.3	
							CO_2 & temperature	1	+325 µmol/mol & +4 ºC		-11.9	
31	Costello & Patterson 2012	Regulated deficit irrigation effect on yield and wine color of cabernet sauvignon in central California	Field	USA	Grape	Berry	Water availability	8	-75, -50 %ETc	Yield	-35.8, +24.3	5
32	De Rosas et al 2017	Loss of anthocyanins and modification of the anthocyanin profiles in grape berries of Malbec and Bonarda grown under high temperature conditions	Field	Argentina	Grape	Berry	Temperature	35	+2.75 ºC	Flavonoids	-71.4, +896.3	5
33	DeGaris et al 2015	Impact of deficit irrigation strategies in a saline environment on Shiraz yield, physiology, water use and tissue ion concentration	Field	Australia	Grape	Berry	Water availability	3	-155, -48 mm	Yield	-25.7, -6.4	4
34	Diaz et al 2016	Water stress versus yield components and quality must (Vitis vinifera L.) cv. Negra Criolla	Field	Peru	Grape	Berry	Water availability	2	-10, -5 L/plant	Yield	-13.3, 0.0	4
								2	-10, -5 L/plant	Phenols	-4.0, -3.4	
								2	-10, -5 L/plant	Flavonoids	-16.7, -1.6	
35	Dos Santos et al 2007	Effects of deficit irrigation strategies on cluster microclimate for improving fruit composition of Moscatel field-grown grapevines	Field	Portugal	Grape	Berry	Water availability	2	-100, -50 %ETc	Yield	-19.8, +0.7	4
								2	-100, -50 %ETc	Phenols	+3.9, +13.0	
36	Drogoudi & Ashmore 2002	Effects of elevated ozone on yield and carbon allocation in strawbetty cultivars differing in developmental stage	Field	UK	Strawberry	Berry	O_3	1	+37.6 ppb	Yield	-40.7%	5

37	Edwards & Clingeleffer 2013	Interseasonal effects of regulated deficit irrigation on growth, yield, water use, berry composition and wine attributes of Cabernet Sauvignon grapevines	Field	Australia	Grape	Berry	Water availability	6	-729, -531 mm	Yield	-12.2, -7.9	4
38	Egea et al 2010	Agronomic response and water productivity of almond trees under contrasted deficit irrigation regimes	Field	Spain	Almond	Nut	Water availability	12	-522, -138 mm	Yield	-34.8, -9.7	4
39	Egea et al 2013	Almond agronomic response to long-term deficit irrigation applied since orchard establishment	Field	Spain	Almond	Nut	Water availability	15	-524, -123, mm	Yield	-52.1, -3.9	4
40	El Jaouhari et al 2018	Assessment of sustainable deficit irrigation in a Moroccan apple orchard as a climate change adaptation strategy	Field	Morocco	Apple	Pome	Water availability	6	-266, -109 Lm^{-2}	Yield	-21.5, +55.9	4
41	Galli et al 2016	Mild salt stress improves strawberry fruit quality	Greenhouse	Brazil	Strawberry	Berry	Water salinity	2	+0.3, +0.7 dS/m	Yield	-7.6, +4.6	4
							Water salinity	2	+0.3, +0.7 dS/m	Antioxidants	+3.9, +12.6	
							Water salinity	2	+0.3, +0.7 dS/m	Phenols	+2.8, +13.8	
							Water salinity	2	+0.3, +0.7 dS/m	Flavonoids	+23.0, +59.8	
42	García-Sánchez et al 2003	Effects of salinity and rate of irrigation on yield, fruit quality and mineral composition of 'Fino 49' lemon	Field	Spain	Lemon	Citrus	Water availability	3	-25 %ETc	Yield	-25.8, -7.3	4
							Water salinity	6	+1.5, +3 ds/m	Yield	-94.2, +5.6	
43	García-Tejero et al 2011	Long-Term impact of sustained-deficit irrigation on yield and fruit quality in sweet orange cv. Salustiana (SW Spain)	Field	Spain	Orange	Citrus	Water availability	12	-50, -25 %ETc	Yield	-20.7, +2.2	4
44	García-Tejero et al 2011	Benefits of low-frequency irrigation in citrus orchards	Field		Orange	Citrus	Water availability	3	-40 %ETc	Yield	-30.0, -26.5	4

45	Garrot et al 1993	Production, growth, and nut quality in pecans under water stress based on the crop water stress index	Field	USA	Pecan	Nut	Water availability	11	=1067, -127 mm	Yield	-23.2, +54.5	4
46	Gehrmann et al 1985	Growth, yield and fruit quality of strawberries as affected by water supply	Greenhouse	Germany	Strawberry	Berry	Water availability	3	-75, -25 %	Yield	-78.9, -53.1	4
47	Gohari et al 2018	Estimate of peanut production function under irrigated conditions and salinity	Field	Iran	Peanut	Nut	Water availability	6	-117, -36 mm	Yield	-24.2, +13.3	4
							Water Salinity	6	+2, +6 dS/m		-27.1, +4.7	
							Water availability & salinity	18	-117, -36mm & +2, +6 dS/m		-43.6, -6.2	
48	Golombek et al 2001	Effect of separate pod and root zone temperatures on yield and seed composition of three Spanish cultivars of groundnut (Arachis hypogaea L)	Greenhouse	India	Peanut	Nut	Temperature	3	+12 ºC	Yield	-51.6, -17.6	4
49	Gonzalez-Dugo et al 2018	The impact of deficit irrigation on transpiration and yield of mandarin and late oranges	Field	Spain	Orange	Citrus	Water availability	16	-291, -63 mm	Yield	-51.3, +12.9	4
50	Hamdam et al 2017	The effect of partial root drying and regulated deficit irrigation technique on growth of rock melon (Cucumis meol linn cv. Glamour)	Rain shelter	Malaysia	Melon	Cucurbits	Water availability	1	-50% ETc	Yield	-28.1	4
51	Han et al 2012	Effects of elevated carbon dioxide and temperature on photosynthesis and fruit characteristics of 'Nikita' pear (Pyrus pyrifolia Nakai)	Greenhouse	Korea	Pear	Pome	CO_2	1	+310 ppm	Yield	+16.6	4
							Temperature	1	+4 ºC	Yield	-15.3	
							CO_2 & temperature	1	+310 ppm & +4 ºC	Yield	+11.9	
52	Haro et al 2008	Seed yield determination of peanut crops under water deficit: Soil strength effects on pod set, the source-sink ratio and radiation use efficiency	Field	Argentina	Peanut	Nut	Water availability	4	-54 %	Yield	-92.6, -73.8	4

53	Heryani et al 2016	Production and quality enhancement of mango using fan jet sprayer irrigation technique	Field	Indonesia	Mango	Drupe	Water availability	3	-100, -25 %ETc	Yield	-0.9, +81.0	4
54	Intrigliolo et al 2016	Effects of post-varaison irrigation regime on Cabernet Sauvignon grapevines in Valencia, Spain: Yield and grape composition	Field	Spain	Grape	Berry	Water availability	9	-75, -25 % ETc	Yield	-26.5, +10.1	4
55	Josuttis et al 2011	Effects of air and soil temperatures on the chemical composition of fruit and agronomic performance in strawberry (Fragaria X ananassa Duch.)	Greenhouse	Germany	Strawberry	Berry	Temperature	1	+6 ºC	Vitamin C	-12.2	4
							Temperature	1	+6 ºC	Antioxidants	+22.9	
							Temperature	1	+6 ºC	Flavonoids	+10.5, +70.6	
56	Junquera et al 2012	Long-term effects of different irrigation strategies on yield components, vine vigour, and grape composition in cv. Cabernet-Sauvignon (Vitis vinifera L.)	Field	Spain	Grape	Berry	Water availability	10	-339, -84 mm	Yield	-83.5, +25.5	4
57	Kapur et al 2018	Irrigation regimes and bio-stimulant application effects on yield and morpho-physiological responses of strawberry	Field	Turkey	Strawberry	Berry	Water availability	2	-50, -35 %Epan	Yield	-36.9, +3.2	4
58	Keshavarzpour & Rashidi 2011	Response of crop yield and yield components of cantaloupe to drought stress	Field	Iran	Cantaloupe melon	Cucurbits	Water availability	3	-60, -20 %	Yield	-40.7, -8.5	4
59	Kirnak & Dogan 2009	Effect of seasonal water stress imposed on drip irrigated second crop watermelon grown in semi-arid climatic conditions	Field	Turkey	Watermelon	Cucurbits	Water availability	8	-100, -25 %	Yield	-80.9. -13.9	4
60	Kirnak et al 2003	Effects of preharvest drip-irrigation scheduling on strawberry yield, quality and growth	Field	Turkey	Strawberry	Berry	Water availability	8	-100, -25%ETc	Yield	-60, -5	4
61	Kizildeniz et al 2015	Effects of climate change including elevated CO2 concentration, temperature and water deficit on growth, water status, and yield quality	Greenhouse	Spain	Grape	Berry	CO_2	2	+300 µmol/mol	Yield	-23.3, 5.6	4
							Temperature	2	+4 ºC	Yield	-19.7, -6.1	

		of grapevine (Vitis vinifera L.) cultivars					CO_2 & Temperature	2	+300 µmol/mol & +4 ºC	Yield	-7.0, +2.1	
							CO_2 & Temperature	1	+300 µmol/mol & +4 ºC	Flavonoids	+50.8	
62	Klein et al 2015	Interacting effects of pollination, water and nutrients on fruit tree performance	Field	USA	Almond	Nut	Water availability	1	-81 L/3 days	Yield	-43.9	5
63	Le Miere et al 1998	The effect of thermal environment, planting data and crown size on growth, development and yield of Fragaria x ananassa Duch. Cv. Elsanta	Greenhouse	UK	Strawberry	Berry	Temperature	4	+4 ºC, +16 ºC	Yield	-84.5, -35.1	4
64	Ledesma et al 2008	Effect of high temperature stress on the reproductive growth of strawberry cvs. 'Nyoho' and 'Toyonoka'	Greenhouse	Japan	Strawberry	Berry	Temperature	4	+7 ºC	Yield	-60.0, -29.6	4
65	Lepaja et al 2016	Influence of regulated deficit irrigation and mulch in reduction vegetative growth and increase fruit yield on Williams pear	Field	Kosovo	Pear	Pome	Water availability	3	-60, -20 %Etc	Yield	-41.8, +19.2	4
66	Leskovar et al 2007	Environmental and genetic factors on carotenoids and quality in watermelon fruits	Field	USA	Watermelon	Cucurbits	Water availability	6	-50, -25 %ETc	Yield	-36.3, -11.6	4
							Water availability	4	-50, -25 %ETc	Carotenoids	-9.3, +5.1	
67	Mabhaudhi et al 2013	Growth, phenological and yield responses of a bambara groundnut (Vigna subterranea L. Verdc) landrace to imposed water stress: II. Rain shelter conditions	Rain shelter	South Africa	Barambara groundnut	Nut	Water availability	2	-70, -40 %	Yield	-93.8, -57.5	4
68	Martínez-Ferri et al 2016	Water relations, growth and physiological response of seven strawberry cultivars (Fragaria × ananassa Duch.) to different water availability	Greenhouse	Spain	Strawberry	Berry	Water availability	7	-50 %Etc	Yield	-59.7, -15.0	4
69	Martinez-Luscher et al 2014	Effects of UV-B radiation on grapevine (Vitis vinifera cv. Tempranillo) leaf physiology and berry composition, framed within the climate	Greenhouse	Spain	Grape	Berry	CO_2 & Temperature	4	+300 ppm, +4 ºC	Carotenoids	-8.2, +6.3	5

		change scenario (water deficit, elevated CO2 and elevated temperature)					CO_2 & Temperature	2	+300 ppm, +4 ºC	Flavonoids	-77.4, -6.5	
70	Masan et al 2018	Effects of irrigation and fertigation on yield and quality parameters of 'Gala' and 'Fuji' apple	Field	Slovakia	Apple	Pome	Water availability	2	-306.8, -265.8 mm	Yield	-3.4	4
71	Mavrogianopoulos et al 1999	Effect of carbon dioxide enrichment and salinity on photosynthesis and yield in melon	Greenhouse	Greece	Melon	Cucurbits	CO_2	2	+400, +800 µmol/mol	Yield	+5.6, 28.3	4
72	Mehdi-Tounsi et al 2017	Long-term field response of pistachio to irrigation water salinity	Field	Tunisia	Pistachio	Nut	Water Salinity	12	+3.1, +10.1 dS/m	Yield	-100.0, +5500.0	4
73	Mena et al 2013	Sustained deficit irrigation affects the colour and phytochemical characteristics of pomegranate juice	Field	Spain	Pomegranate	Pome	Water availability	4	-63, -32 %ETc	Flavonoids	-75.1, +10.3	4
74	Mendlinger et al 1993	Flowering, vegetative growth, yield, and fruit quality in muskmelons under saline conditions	Field	Israel	Muskmelon	Cucurbits	Water Salinity	16	+1.78, +3.66 dS/m	Yield	-24.7, +4.1	4
75	Menzel et al 1995	Water deficits at anthesis reduce CO2 assimilation and yield of lychee (Litchi chinensis Sonn.) trees	Field	South Africa	Lychee	Drupe	Water availability	1	-100 %	Yield	-85.6	4
76	Miras-Avalos et al 2017	Water versus sink:source relationships in a semiarid Tempranillo vineyard: vine performance and fruit composition	Field	Spain	Grape	Berry	Water availability	2	-25 %ETc	Yield	-5.7, -3.9	4
							Water availability	2	-25 %ETc	Phenols	+4.5, +6.7	
							Water availability	2	-25 %ETc	Flavonoids	-5.1, +9.1	
77	Mortley et al 2004	Temperature influences yield, reproductive growth, harvest index, and oil content of hydroponically grown 'Georgia Red' peanut plants	Growth chamber	USA	Peanut	Nut	Temperature	3	+4, +12 ºC	Yield	+476.5, +1347.1	4

78	Moutinho-Pereira et al 2009	Effects of elevated CO2 on grapevine (Vitis vinifera L.): physiological and yield attributes	Field	Portugal	Grape	Berry	CO_2	3	+135 ppm	Yield	+27.2, +50.3	5
79	Myers et al 1995	Long term effects of saline irrigation on the yield and growth of mature Williams pear trees	Field	Australia	Pear	Pome	Water salinity	41	+0.5, +1.9 dS/m	Yield	-55.3, +31.0	4
80	Pedrero et al 2018	Use of reclaimed wastewater on fruit quality of nectarine in Southern Italy	Field	Italy	Nectarine	Citrus	Water salinity	1	+1 dS/m	Yield	-2.8	5
							Water salinity	1	+1 dS/m	Phenols	+10.4	
							Water salinity	1	+1 dS/m	Antioxidants	+2.7	
81	Perez-Perez et al 2016	Comparison of deficit and saline irrigation strategies to confront water restriction in lemon trees grown in semi-arid regions	Field	Spain	Lemon	Citrus	Water availability	1	-75 %Etc	Yield	-34.3	4
82	Perez-Sarmiento et al 2016	Effects of regulated deficit irrigation on physiology, yield and fruit quality in apricot trees under Mediterranean conditions	Field	Spain	Apricot	Drupe	Water availability	1	-149.6 mm	Yield	-1.69	4
83	Prasad et al 2003	Super-optimal temperatures are detrimental to peanut (Arachis hypogaea L.) reproductive processes and yield at both ambient and elevated carbon dioxide	Growth chamber	USA	Peanut	Nut	CO_2	1	+350 µmol/mol	Yield	+21.0	4
							Temperature	3	+4, +12 °C		-90.1, -14.1	
							CO_2 & Temperature	3	+350 µmol/mol & +4, +12 °C		-85.9, +2.7	
84	Redalen et al 1993	Fruit and shoot development in apple as affected by temperature, light, humidity and a possible greenhouse effect in simulated latitudes	Greenhouse	Sweden	Apple	Pome	Temperature	1	+2 °C	Vitamin C	-81.9	4
85	Retzlaff et al 1997	Growth and yield response of commercial bearing-age ‘Casselman’plum trees to various ozone partial pressures	Field	USA	Plum	Drupe	O_3	2	+0.04, +0.045 µPa/Pa	Yield	-57.3, -13.4	5
86	Roccuzzo et al 2016	Deficit irrigation for enhancing 'Tarocco' orange fruit quality	Field	Italy	Orange	Citrus	Water availability	2	-50, -30% ETc	Yield	-24.3, -7.0	5

							Water availability	2	-50, -30% ETc	Vitamin C	+4.7, +6.0	
							Water availability	2	-50, -30% ETc	Flavonoids	+23.5, +52.6	
87	Romero et al 2004	Effects of regulated deficit irrigation during the pre-harvest period on gas exchange, leaf development and crop yield of mature almond trees	Field	Spain	Almond	Nut	Water availability	2	-161.8 mm	Yield	-16.3, -3.7	4
88	Sadras & Moran 2013	Asymmetric warming effect on the yield and source:sink ratio of field-grown grapevine	Field	Australia	Grape	Berry	Temperature	2	+1.6 ºC	Yield	-16.9, +104	5
89	Selahvarzi et al 2017	Effect of deficit irrigation on flowering and fruit properties of pomegranate (Punica granatum cv. Shahvar)	Field	Iran	Pomegranate	Pome	Water availability	2	-50 %Etc	Yield	-52.1, -36.1	4
90	Sharma et al 2014	Root growth, yield and fruit quality responses of reticulatus and inodurus melons (Cucumis melo L.) to deficit subsurface drip irrigation	Field	USA	Melon	Cucurbits	Water availability	6	-50 %ETc	Yield	-42.9, -12.3	4
							Water availability	4	-50 %ETc	Vitamin C	-11.4, +90.5	
							Water availability	2	-50 %ETc	Carotenoids	+0.9, +15.4	
91	Sharma et al 2016	Differential response of muskmelon (Cucumis melo L.) cultivars to deficit irrigation	Field	USA	Melon	Cucurbits	Water availability	6	-50 %ETc	Yield	-43.3, -16.6	4
							Water availability	6	-50 %ETc	Vitamin C	-13.9, +117.9	
							Water availability	4	-50 %ETc	Carotenoids	-0.5, +23.1	
92	Silvestre et al 2012	Influence of timing and intensity of deficit irrigation on vine vigour, yield and berry and wine composition of 'Tempranillo' in S. Portugal	Field	Portugal	Grape	Berry	Water availability	6	-130, -10 mm	Flavonoids	-20.4, +7.7	4

93	Sofo et al 2012	Berry morphology and composition in irrigated and non-irrigated grapevine (Vitis vinifera L.)	Field	Italy	Grape	Berry	Water availability	1	-100 %ETc	Yield	-33.3	4
							Water availability	1	-100 %ETc	Flavonoids	+28.3	
94	Spreer et al 2009	Yield and fruit development in mango (Mangifera indica L. cv. Chok Anan) under different irrigation regimes	Field	Thailand	Mango	Drupe	Water availability	6	-100, -50 %ETc	Yield	-27.1, +48.4	4
95	Stagno et al 2015	Response of orange trees to deficit irrigation strategies: Effects on plant nutrition, yield and fruit quality	Field	Italy	Orange	Citrus	Water availability	6	-50, -30 %ETc	Yield	-49.1, +10.6	4
96	Stanciel et al 2000	Growth, pod, and seed yield, and gas exchange of hydroponically grown peanut in response to CO2 enrichment	Growth chamber	USA	Peanut	Nut	CO_2	2	+400, +800 µmol/mol	Yield	+55.9, +61.9	4
97	Sun et al 2012	Effects of elevated CO2 and temperature on yield and fruit quality of Strawberry (Fragaria ananassa Duch.) at 2 levels of nitrogen application	Greenhouse	China	Strawberry	Berry	CO_2	1	+360 ppm	Yield	+133.4	4
							CO_2	1	+360 ppm	Phenols	-21.2	
							CO_2	2	+360 ppm	Flavonoids	-48.1, -36.1	
							Temperature	1	+5 ºC	Yield	+22.3	
							CO_2 & Temperature	1	+360 ppm & +5 ºC	Yield	+8.8	
							CO_2 & Temperature	1	+360 ppm & +5 ºC	Phenols	-19.6	
							CO_2 & Temperature	2	+360 ppm & +5 ºC	Flavonoids	-35.8, -30.1	
98	Sung & Chen 1991	Gas exchange rate and yield response of strawberry to carbon dioxide enrichment	Greenhouse	Taiwan	Strawberry	Berry	CO_2	1	+660 µmol/mol	Yield	+43.4	4
99	Tarricone et al 2017		Field	Italy	Grape	Berry	Water availability	1	-20 %ETc	Yield	-6.6	4

		"Grape and wine quality of Vitis vinifera 'Nero di Troia' in response to moderate deficit irrigation"					Water availability	1	-20 %ETc	Phenols	+13.2	
							Water availability	2	-20 %ETc	Flavonoids	+5.3, +10.6	
100	Terry et al 2007	Effect of water deficit irrigation and inoculation with Botrytis cinerea on strawberry (Fragaria x ananassa) fruit quality	Greenhouse	UK	Strawberry	Berry	Water availability	2	-150, -100 ml/day	Vitamin C	+9.9, +11.3	4
							Water availability	2	-150, -100 ml/day	Antioxidants	+1.5, +12.0	
101	Tripathi et al 2013	Interactive effect of supplemental ultraviolet B and elevated ozone on seed yield and oil quality of two cultivars of linseed (Linum usitatissimum L.) carried out in open top chambers	Open top chamber	India	Linseed	Seed	O_3	2	+10 ppb	Yield	-43.2, -22.9	5
102	Yang et al 2018	Fruit yield and quality response of Newhall navel orange to different irrigation regimes and ground cover in Chongqing Three Gorges Reservoir area	Field	China	Orange	Citrus	Water availability	2	-50, -110 %ETc	Yield	+31.3, +56.3	4
							Water availability	2	-50 %ETc	Vitamin C	+13.8, +17.8	
103	Zhang et al 2017	Biophysical response of young pomegranate trees to surface, sub-surface and drip irrigation and deficit irrigation	Field	USA	Pomegranate	Pome	Water availability	6	-65, -25 %	Yield	-34.4, +3.5	4
104	Zhou et al 2017	Peach yield and fruit quality is maintained under mild deficit irrigation in semi-arid China	Field	China	Peach	Drupe	Water availability	6	-75, -25 %ETc	Yield	-42.5, +0.4	4
							Water availability	6	-75, -25 %ETc	Vitamin C	+3.2, +22.5	
105	Zong et al 2011	Effect of different irrigation water salinities on some yield and quality components of two field-grown Cucurbit species	Field	China	Melon	Cucurbits	Water salinity	2	+1.24, +4.95 dS/m	Yield	-26.9, -4.4	5
					Watermelon		Water salinity	2	+1.24, +4.95 dS/m	Yield	-51.5, -18.8	
